# A novel fatty acid metabolism-related gene prognostic signature and candidate drugs for patients with hepatocellular carcinoma

**DOI:** 10.7717/peerj.14622

**Published:** 2023-01-06

**Authors:** Jingze Yang, Xin Yang, Jinlu Guo, Shi Liu

**Affiliations:** Gastroenterology, Union Hospital, Tongji Medical College, Huazhong University of Science and Technology, Wuhan, Hubei, China

**Keywords:** Fatty acid metabolism, Prognosis, Immune infiltration, Drug sensitivity, Hepatocellular carcinoma

## Abstract

Hepatocellular carcinoma (HCC) is one of the deadliest cancers. Fatty acid metabolism (FAM) is associated with the development and treatment of HCC. This study aimed to build a FAM-related gene model to assess the prognosis of HCC and provide guidance for individual treatment. RNA-sequencing data of patients with HCC from The Cancer Genome Atlas and Gene Expression Omnibus database (GSE14520) were extracted as the training and validation sets, respectively. A FAM-related gene predictive signature was built, and the performance of prognostic model was assessed. The immune infiltration and drug sensitivity were also evaluated. Quantitative real-time polymerase chain reaction and western blot were performed to evaluate the levels of the model genes. A 12-gene FAM-related risk signature was constructed; patients with a higher risk score had poorer prognosis than those with a lower risk score. Risk score was shown as an independent risk factor for overall survival of HCC, and the signature was further confirmed as an effective and accurate model. A nomogram was constructed, and it exhibited the good performance in the prognostic prediction. In addition, the immune cell infiltration and sensitivity to chemotherapy drugs were correlated with different risk levels. Finally, quantitative real-time polymerase chain reaction and western blot proved the changes of above genes. Differential expression of FAM-related genes can be used to predict response to immunotherapy and chemotherapy, and improve the clinical prognosis evaluation of patients with HCC, which provides new clues for further experimental exploration and verification on FAM-related genes in HCC.

## Introduction

Liver cancer is reported to be the third primary reason of cancer-related death worldwide, only behind lung and colorectal cancers (Sung et al., 2021). Notably, hepatocellular carcinoma (HCC) accounts for 90% of primary liver cancers (Forner, Reig & Bruix, 2018). Presently, HCC treatment mainly concludes surgery, chemotherapy, immunotherapy, and radiotherapy, but the actual long-term survival rate of HCC patients is still unsatisfactory (Gluer et al., 2012). Studies showed the genomic and transcriptional heterogeneity among patients with HCC may be associated with relapse, drug resistance, and poor prognosis (Xu et al., 2019). Therefore, valid biomarkers are needed for individualized treatment and prognostic prediction in patients with HCC.

Metabolic rewiring can disrupt cellular homoeostasis, cause excessive cell growth and proliferation, and further promote cancer. Changes in lipid metabolism acclimate cancer cells to the various microenvironment by resisting oxidative stress, adjusting intercellular communication, regulating immune responses, and sustaining key oncogenic functions (Butler et al., 2020). Therefore, lipid profiles are emerging as the biomarkers for the prognostic prediction in patients with cancer. Zhu et al. (2021) constructed a model on the basis of lipid metabolism-related genes and verified its good performance for prognostic prediction in patients with bladder cancer. Abnormal fatty acid (FA) metabolism (FAM) in cancer has attracted increased attention as it was recently shown to affect cancer cell biology (Koundouros & Poulogiannis, 2020). FA not only regulates the synthesis of biological membranes, but also takes part in the modulation of oncogenic signaling. In addition, FA serves as substrates for mitochondrial energy synthesis and the form of energy storage (Hoy, Nagarajan & Butler, 2021). Wang et al. (2016a) revealed the promotion of FAM in maintenance of HCC cell survival. Therefore, targeting the FAM may be an underlying method for the HCC treatment. However, due to the diversity of genotypes and oncobiology, it is difficult to stratify patients with HCC, which limits drug discovery based on the FAM. Notably, the development of transcriptomics and metabolomics reveal a novel approach to HCC grouping, and may provide clinical guidance targeting FAM. Ding et al. (2021) found prognostic model in colorectal cancer via FAM-related genes and exhibited that low-risk patients were more sensitive to chemotherapeutic agents. Consequently, targeting the FAM may contribute to the development of a novel signature for the prognostic prediction and clinical medication for patients with HCC.

Here, we aimed to construct a FAM-related gene model to measure the risk scores, improve the accuracy of prognosis prediction, explore the drug sensitivity and immune cell infiltration, and further provide the better guidance for clinical treatment of patients with HCC.

## Materials & Methods

### Data acquisition

The raw RNA sequencing data of HCC were acquired from The Cancer Genome Atlas (TCGA) database on March 14, 2022. Meanwhile, the clinical information was also acquired. The GSE14520 (GPL3921) dataset, including data about gene levels and clinical features of patients with HCC, was extracted from Gene Expression Omnibus (GEO) database. Notably, samples with incomplete clinical data were excluded. Finally, 370 samples with HCC from the TCGA database were applied as the training set and 221 samples with HCC from the GEO database were used as the validation set.

### Selecting FAM-related differentially expressed genes

A total of 309 FAM-related genes were extracted in FAM pathways, and then 137 common genes in TCGA and GEO cohorts were identified (Table S1). Moreover, the differentially expressed FAM-related genes (DEFAMGs) between patients with HCC and control individuals were obtained on the basis of the following criteria: False Discovery Rate <0.05 and log FC = 0.585. Then, Gene Ontology (GO) and Kyoto Encyclopedia of Genes and Genomes (KEGG) enrichment analyses were carried out on DEFAMGs using the “cluster Profiler” R package.

### Constructing and validating the FAM-related prognostic model

In the training set, the genes significantly related to patient overall survival (OS) were selected from DEFAMGs using the Univariate Cox analysis. *P* value <0.01 was regarded as significant. To avoid overfitting, least absolute shrinkage and selection operator (LASSO) regression analysis was adopted to construct a prognostic model (Liang et al., 2020). With the “glmnet” R package, the LASSO algorithm was utilized for variable selection and shrinkage. The independent variable in the regression was the normalized expression matrix of candidate prognostic DEFAMGs, and the response variables in the training set were OS and patients’ status. According to each gene’s normalized expression and related regression coefficients, the risk score of each patient was computed. The formulae for these calculations have been previously described (Zhao et al., 2021). Then, Kaplan–Meier (K-M) analysis and generation of receiver operating characteristic (ROC) curve were carried out. Next, the independent prognostic performance of the model was assessed by regression analysis. Besides, the principal-component analysis on the basis of FAM-related genes and genes used to build prognostic signature model was performed using the “limma” and “ggplot2” R packages. Finally, the FAM-related prognostic signature was further tested using the K-M analysis and ROC curve in the validation set (GSE14520).

### Establishing a predictive nomogram

The nomogram model was created to forecast the OS for patients with HCC via the “rms”, “regplot”, and “survival” packages. Then, the prognostic performance of the nomogram was assessed from various aspects.

### Exploration of the model in immunity therapy

The gene sets, which included numerous different types of human immune cell subtypes and immune-related activities, were gathered for evaluation of immune-related characteristics as previously described (Ding et al., 2021). Single-sample gene-set enrichment analysis (ssGSEA) was performed using the “GSEABase” and “GSVA” R packages to observe the immune-related infiltration in patients with HCC. To explore the differences of biological processes between low- and high-risk score groups, GSVA was performed on the gene profile using the “GSVA” R package. The reference gene sets were “c2.cp.kegg.v7.4.symbols” from the Molecular Signatures Database (https://www.gsea-msigdb.org/gsea/msigdb).In addition, Tumor Immune Dysfunction and Exclusion (TIDE) was applied to forecast the feasibility of immunotherapy (Xu et al., 2020). Moreover, the Wilcoxon test was used to test intergroup differences in the levels of potential immune checkpoint molecules, such as PD-1 and CTLA-4. Statistical significance was set at FDR >0.05.

### Exploration of chemotherapy response based on risk model

In order to predict the therapeutic effect of commonly used chemotherapeutic drugs, including sorafenib, gemcitabine, 5-fluorouracil, paclitaxel, and lapatinib, the half-maximal inhibitory concentration (IC50) of chemotherapeutic agents was detected using the “pRRophetic” R package. The relationship between the risk score and IC50 of chemotherapeutic drugs was also assessed.

### Cell culture and real-time polymerase chain reaction

The human hepatocyte cell line MIHA and HCC cell lines, MHCC-97H and Huh-7, were obtained from the BeNa Culture Collection (Suzhou, China), and cultured in Dulbecco’s modified Eagle medium with 10% fetal bovine serum in a 37 °C humidified incubator containing 5% CO2. Total RNA was isolated from cells using the TRI-ZOL reagent (Takara, Otsu, Japan). Then, the cDNA synthesis was performed using the PrimeScript™ RT Master Mix Kit (Takara). Real-time polymerase chain reaction (RT-PCR) was carried out using the SYBR-Green PCR master mix (Takara) in the Roche Light Cycler R480 (Roche, Basel, Switzerland). The Glyceraldehyde-3-phosphate dehydrogenase (GAPDH) was used as the internal reference. The relative expression of mRNA levels was calculated by 2^−ΔΔCt^ and the primer sequences are listed in Table S2.

### Western blot analysis

Proteins were extracted from cells with RIPA lysis buffer (Beyotime, Shanghai, China) containing phenylmethyl sulfonyl fluoride. The concentration of protein was measured by bicinchoninic acid protein assay kit (Beyotime). Then, a suitable quality of protein samples was subjected to sodium dodecyl sulfate polyacrylamide gel electrophoresis and transferred to PVDF membranes (Millipore, Billerica, MA, USA). The 8% skimmed milk was used to block the PVDF membranes at room temperature for 1 h, and the PVDF membranes were incubated with primary antibodies at 4 °C overnight. The corresponding secondary antibodies was used at room temperature for 1 h and the protein signals were detected with FluorChem FC3 system (ProteinSimple, Santa Clara, CA, USA) using an enhanced chemilusystem reagent (Thermo Fisher). Image J was used to measure the gray values of the target and reference bands. Then, the target/ reference ratio was regarded as the relative expression level of target. The antibodies used in this study were as follows: PON1 (1:1000, A3441, Abclonal, Wuhan, China), CYP2C9 (1:4000, GB114267, Servicebio, Wuhan, China), ACACA (1:1000, A19627, Abclonal), ACADS (1:1000, A0945, Abclonal), ME1 (1:1000, A3956, Abclonal), ACAT1 (1:1000, A20943, Abclonal), ELOVL1 (1:1000, A07612, Boster, Wuhan, China), SMS (1:1000, A9380, Abclonal), ADSL (1:1000, A6278, Abclonal), UGDH (1:1000, A1210, Abclonal), HSP90AA1 (1:1000, A5006, Abclonal), S100A10 (1:1000, A13614, Abclonal), GAPDH (Abclonal), horse radish peroxidase (HRP)-linked goat anti rabbit IgG, and HRP-linked goat anti mouse IgG (1:4000, Antgene, Wuhan, China).

### Statistical analysis

The statistical analyses were carried out via R version 4.1.3 (R Core Team, 2021) and SPSS 25.0 (SPSS, Inc., Chicago, IL, USA). The ssGSEA scores of immune cells or pathways between the high- and low-risk groups were compared using the Mann–Whitney test with *P* values corrected using the BH technique. K-M analysis was used to evaluate the OS differences between different groups. Univariate and multivariate Cox regression analyses were performed to identify independent predictors of OS. The Chi-squared test was used to compare proportional differences. Gene and protein levels in different cell lines were analyzed by independent sample *t*-test or correction *t*-test depending on the homogeneity of variance analysis. Statistical significance was set at *P* < 0.05.

## Results

### Enrichment analysis of DEFAMGs

**Figure 1:**
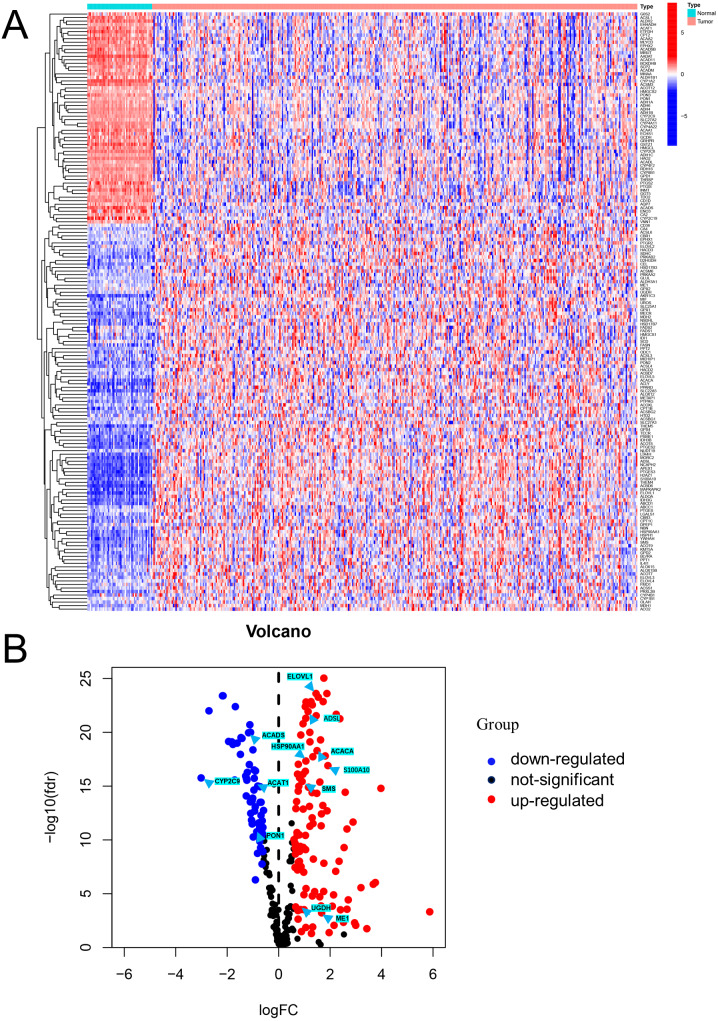
Visualization of differentially expressed FAM-related genes. (A) Heatmap of the differentially expressed FAM-related genes in the low- and high-risk groups. (B) Volcano plot of the differentially expressed FAM-related genes. FAM: fatty acid metabolism.

Differential expression analysis using data from the TCGA database exhibited that there were 170 DEFAMGs, with 110 genes and 60 genes being upregulated and downregulated, respectively in HCC samples (Figs. 1A–1B; Table S3). Then, GO analysis showed that FA metabolic process, long-chain FA metabolic process, and FA biosynthetic process were highly enriched (Figs. S1A–S1B). Meanwhile, the KEGG analysis also exhibited that FAM, degradation, and elongation process were highly concentrated (Figs. S1C–S1D). These findings revealed that FAM played an important part in HCC.

### Building a FAM-related risk signature

**Figure 2:**
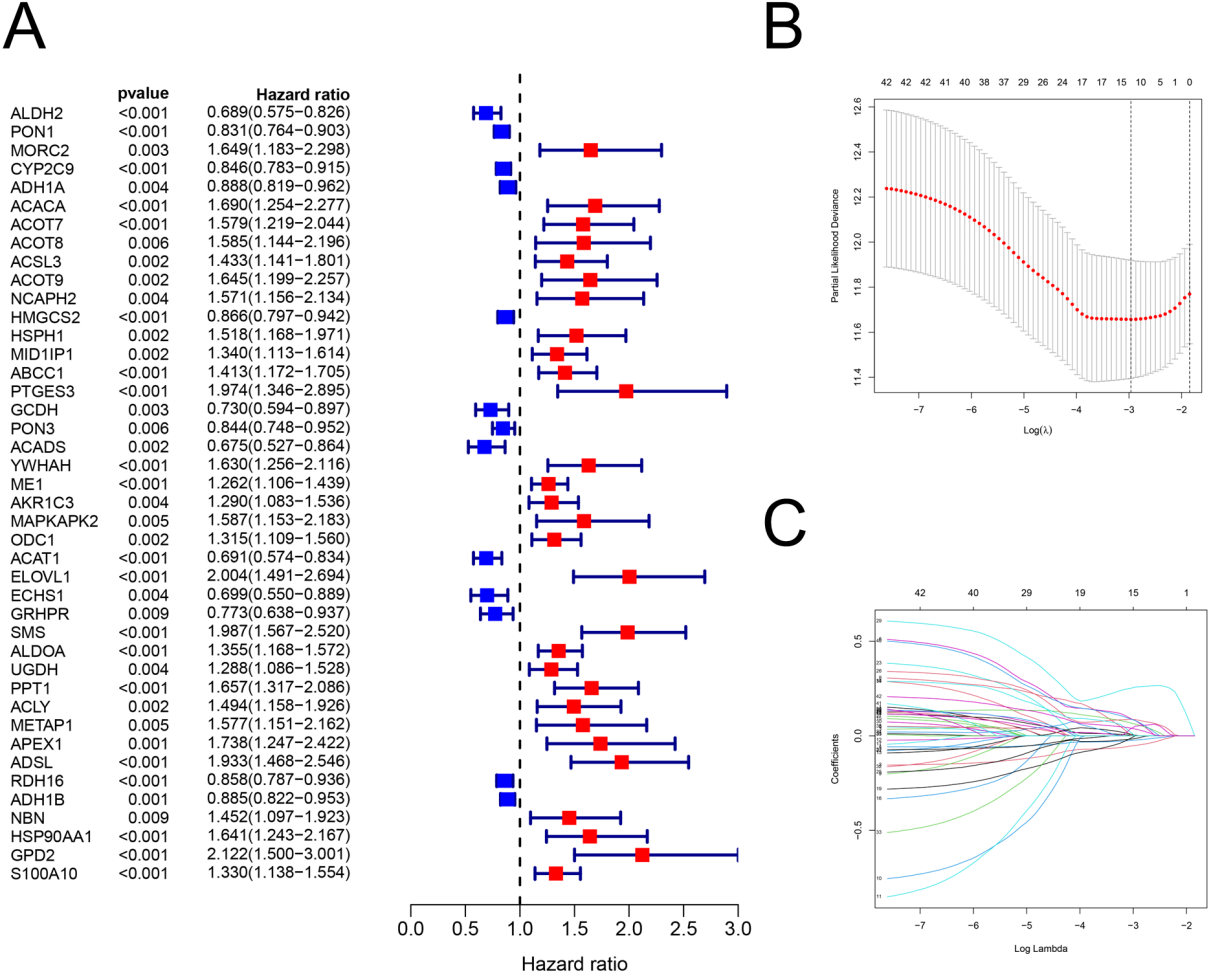
The building of prognostic model. Construction of FAM-related prognostic model. (A) Forest plot displaying the results of the univariate Cox regression analysis between gene expression and OS. (B–C) LASSO regression analysis identified 12 genes that were used to develop the FAM-related prognostic model. FAM, fatty acid metabolism; OS, overall survival; LASSO, least absolute shrinkage and selection operator.

There were 42 genes associated with OS (*P* < 0.01; Fig. 2A; Table S4). After the LASSO regression (Figs. 2B–2C), the following 12 FAM-related genes, PON1, CYP2C9, ACACA, ACADS, ME1, ACAT1, ELOVL1, SMS, UGDH, ADSL, HSP90AA1, and S100A10, were selected to build the prediction model. The formula used was as follows: risk score = (−0.05089 × PON1_expression_) + (−0.0225 × CYP2C9_expression_) + (0.08121 × ACACA_expression_) + (−0.0002 × ACADS_expression_) + (0.06192 × ME1_expression_) + (−0.0144 ×ACAT1_expression_) + (0.08595 × ELOVL1_expression_) + (0.25743 × SMS_expression_) + (0.0033 × UGDH_expression_) + (0.05056 × ADSL_expression_) + (0.08092 × HSP90AA1_expression_) + (0.03604 × S100A10_expression_), which is shown in Table S5.

### Validating the FAM-related risk signature

**Figure 3:**
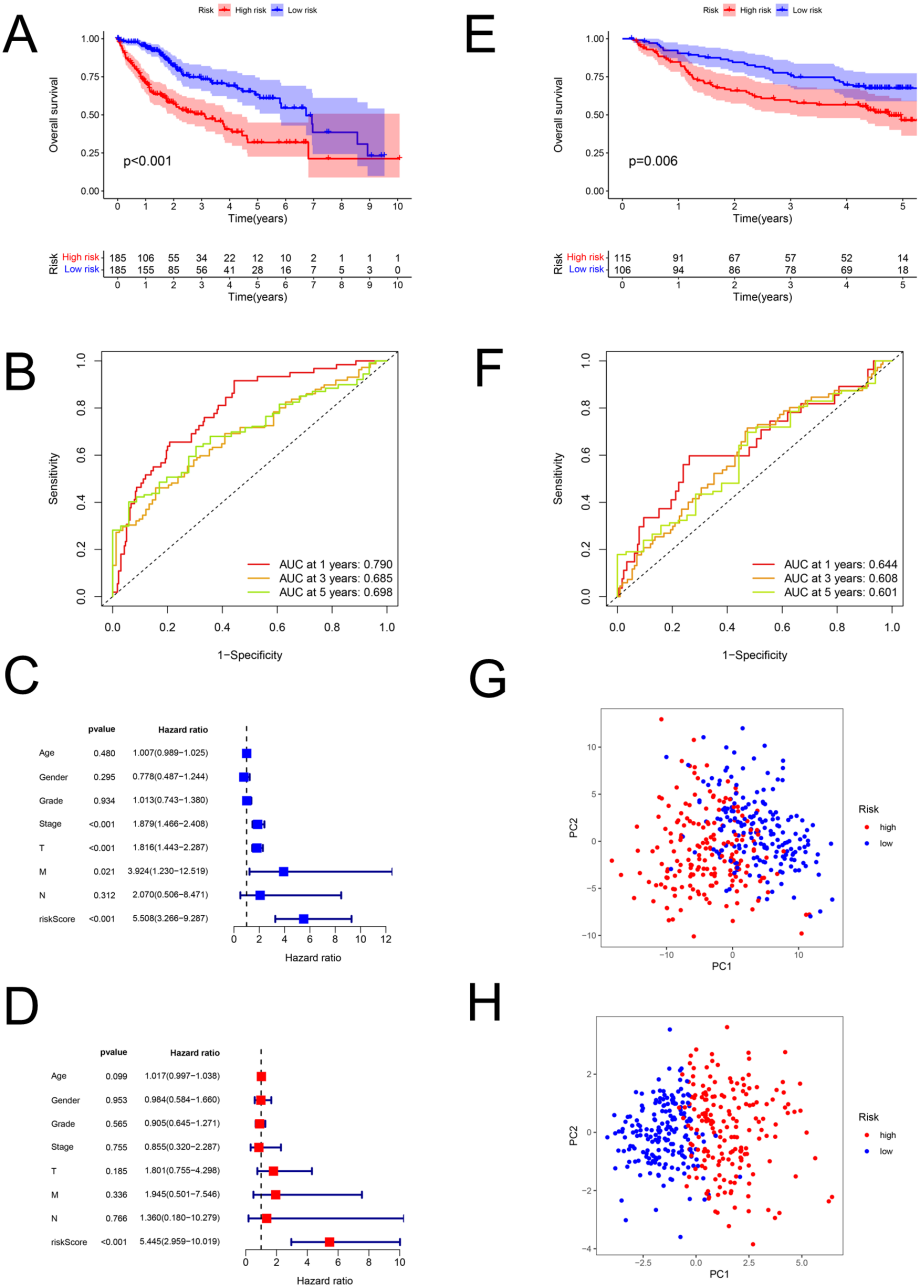
The predictive performance of the model. Predictive performance of the FAM model. (A, E) K-M analysis for the OS of the patients in the low- and high-risk groups in the training and validation sets. (B, F) AUC of time-dependent ROC curves verified the prognostic accuracy of the risk signature at 1-, 3- and 5-year survival time in the training and validation sets. (C, D) Results of the univariate and multivariate Cox regression analyses of OS in the training set. (G, H) Principal-component analysis based on all FAM-related genes and risk genes of the model. FAM, fatty acid metabolism; K-M, Kaplan–Meier; OS, overall survival; AUC, Area under the curve; ROC, receiver operating characteristic.

The risk scores of HCC were computed using the above formula. Using the median risk score, patient samples from the TCGA database were classified into low-risk (*n* = 185) and high-risk (*n* = 185) sets (Table S6). Correspondingly, patients from the GEO database (GSE14520; validation set) were divided into low-risk (*n* = 106) and high-risk (*n* = 115) groups (Table S7). Patients with higher risk had a worse prognosis both in the training (*P* < 0.001; Fig. 3A) and validation (GSE14520) sets (*P* = 0.006; Fig. 3E). In the training set, the AUCs for 1-, 3-, and 5-year survival were 0.790, 0.685, and 0.698, respectively (Fig. 3B), while in the validation set, they were 0.644, 0.608, and 0.601, respectively (GSE14520) (Fig. 3F). The risk score was also verified to be an effective prediction model both in the univariate (*P* < 0.001; Fig. 3C) and multivariate Cox regression (*P* < 0.001; Fig. 3D). Besides, the principal-component analysis displayed that the model had a better performance than FAM-related genes in distinction of the different-risk patients (Figs. 3G–3H).

### Stratification analysis of the prediction model

**Figure 4:**
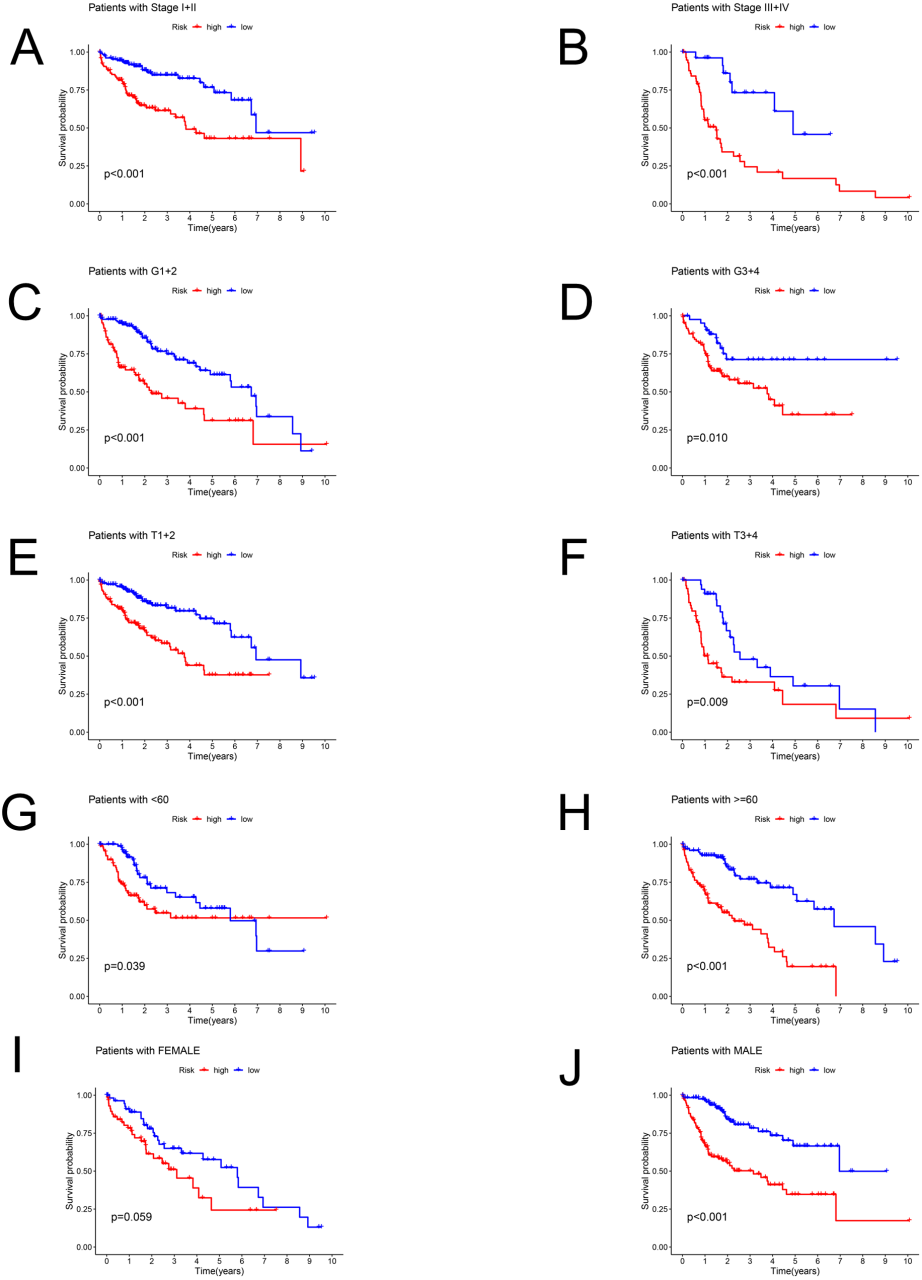
The Kaplan–Meier curve. Kaplan–Meier curves displaying the survival probability in different subgroups. (A) Stage I–II, (B) Stage III–IV, (C) G1+2, (D) G3+4, (E) T1+2, (F) T3+4, (G) age <60 years, (H) age ≥60 years, (I) female sex, (J) male sex.

Patients in the TCGA dataset were separated into several subgroups by different clinical parameters and the OS difference was then explored. We exhibited high-risk patients had a worse prognosis under the condition of Stage I–II (*P* < 0.001), Stage III–IV (*P* < 0.001), G1+2 (*P* < 0.001), G3+4 (*P* = 0.01), T1+2 (*P* < 0.001), T3+4 (*P* = 0.009), age <60 (*P* = 0.039), age ≥ 60 (*P* < 0.001), male (*P* < 0.001) except for female (*P* = 0.059) (Figs. 4A–4J).

### Construction and assessment of a predictive nomogram

**Figure 5:**
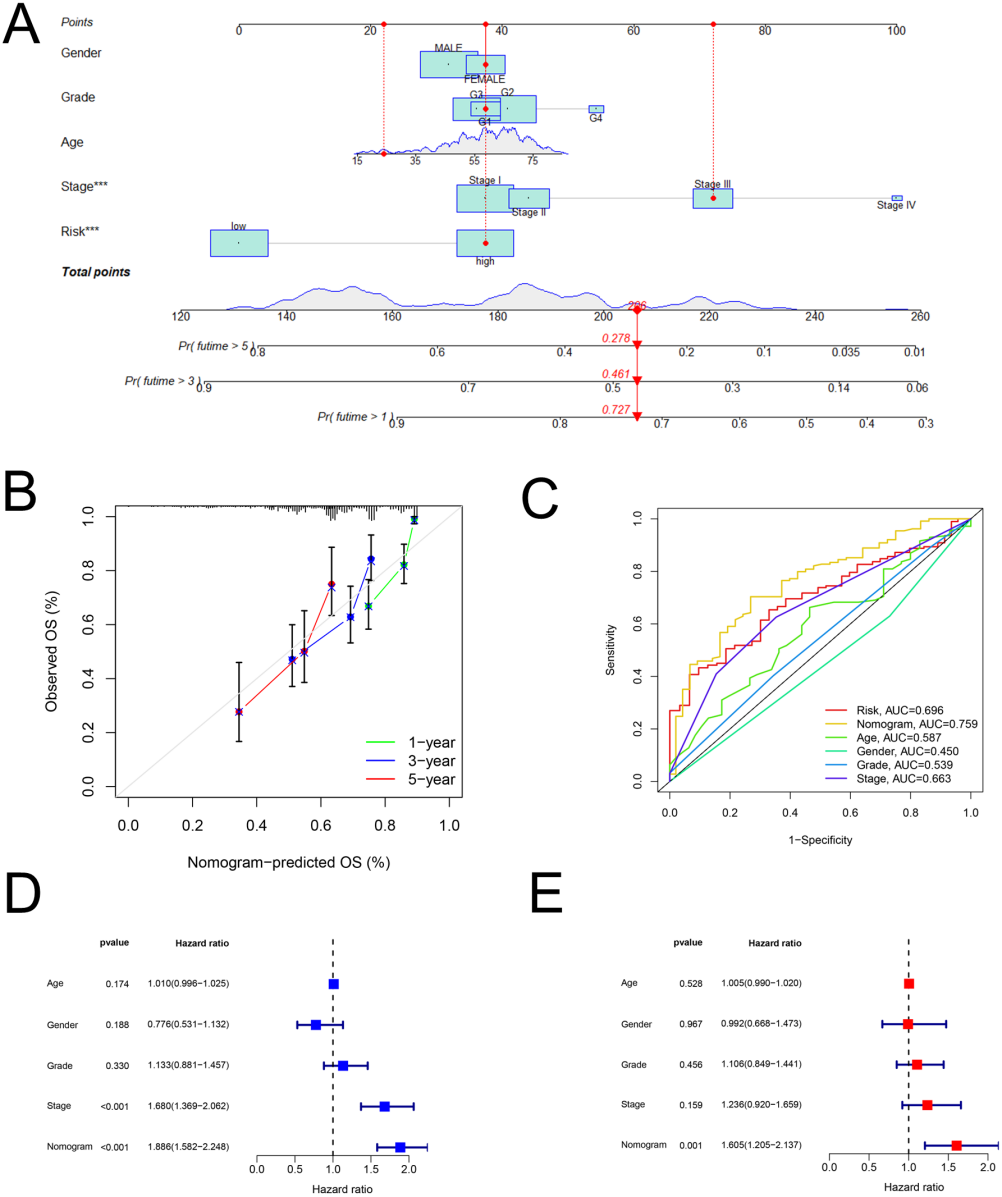
The nomogram. Establishment and evaluation of a nomogram in the training set. (A) Nomograms for predicting the overall survival of patients with HCC at 1-, 3- and 5-year survival time. (B) Calibration curves displaying the concordance between predicted and observed 1-, 3-, and 5-year overall survival. (C) AUC of time-dependent ROC curves verifying the prognostic performance of the nomogram at 5-year overall survival. (D) Results of the univariate Cox regression of OS in the nomogram. (E) Results of the multivariate Cox regression of OS in the nomogram. AUC, Area under the curve; HCC, hepatocellular carcinoma; OS, overall survival; ROC, receiver operating characteristic.

As shown in Fig. 5A, the nomogram was created via clinical information and risk score. Besides, calibration plots exhibited that the observed OS was approximately consistent with the predicted OS (Fig. 5B). Moreover, the nomogram (AUC = 0.759) was illustrated to have better performance in the prediction of OS than a single marker, such as risk score (AUC = 0.696), age (AUC = 0.587), gender (AUC = 0.450), grade (AUC = 0.539), and pathological stage (AUC = 0.663) (Fig. 5C). Then, Figs. 5D and 5E displayed that the nomogram was effective for the prognostic evaluation (*P* < 0.001 and *P* = 0.001, respectively).

### Exploring the immune-related characteristic

**Figure 6:**
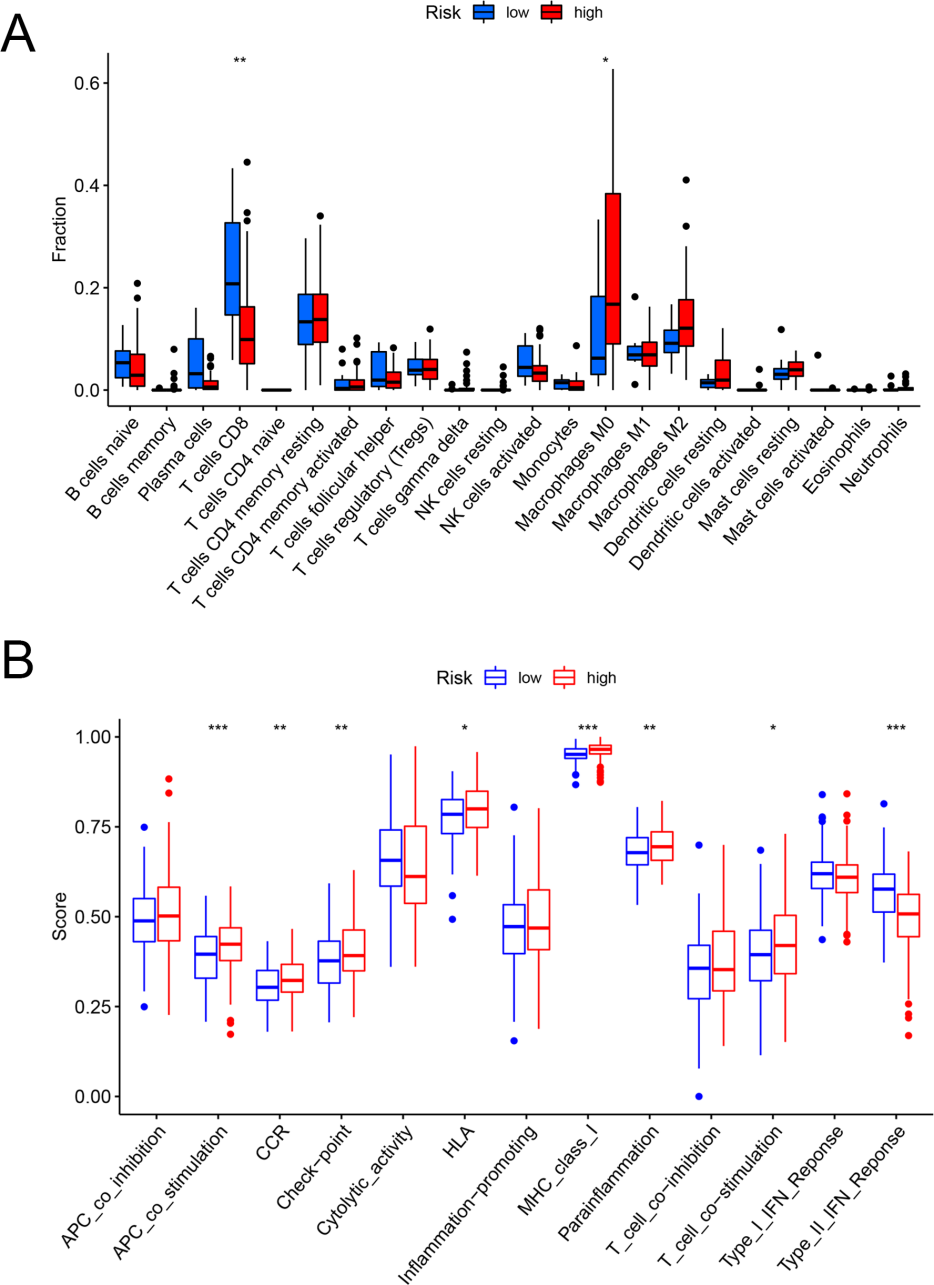
The evaluation of immunological characteristics. Evaluation of immune cells and immunologic function based on risk scores. (A) Fraction of immune cells in the low- and high-risk groups. (B) Immunologic function in the low- and high-risk groups (* *P* < 0.05, ** *P* < 0.01, *** *P* < 0.001.).

Figures 6A and 6B showed that the fractions of CD8+ T cells were higher in the low-risk set, while in the high-risk set, higher fractions were observed in macrophages M0. In addition, as for the immune-related pathways, the scores of APC co-stimulation, CCR, Check-point, HLA, MHC-class-I, para-inflammation, T-cell-co-stimulation in the high-risk scores set was higher than those of the low-risk set. However, the score of Type-II-IFN-Response represented higher activity in the low-risk set.

### Role of the FAM model in predicting the response to immunotherapy

**Figure 7:**
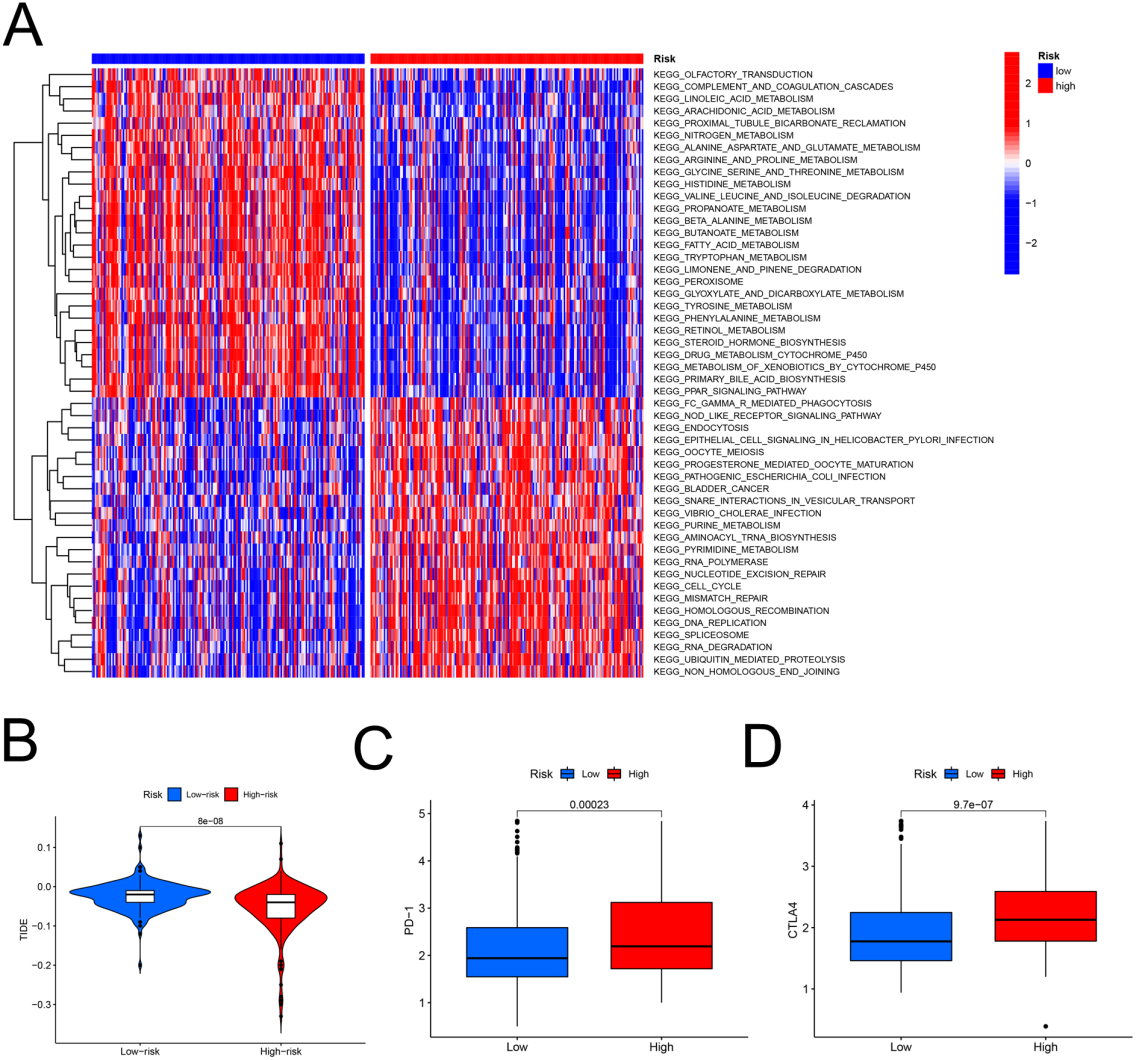
The model in immunotherapy. Ability of the FAM model to predict response to immunotherapy. (A) Heatmap in the low- and high-risk score groups. (B) Comparison of tumor immune dysfunction and exclusion between low- and high-risk groups. (C) Comparison of PD-1 expression between low- and high-risk groups. (D) Comparison of CTLA-4 expression between low- and high-risk groups. FAM, fatty acid metabolism.

The heatmap analysis showed that a majority of metabolic pathways, such as FAM pathway, were intensive in the low-risk set, while the high-risk was associated with the cancer-related and cell signaling-related pathways (Fig. 7A). The TIDE showed higher risk patients may be more suitable for immunotherapy (Fig. 7B). Furthermore, the levels of PD-1 (*P* < 0.001, Fig. 7C) and CTLA-4 (*P* < 0.001, Fig. 7D) were higher in high- than in low-risk patients.

### Response to chemotherapy

**Figure 8:**
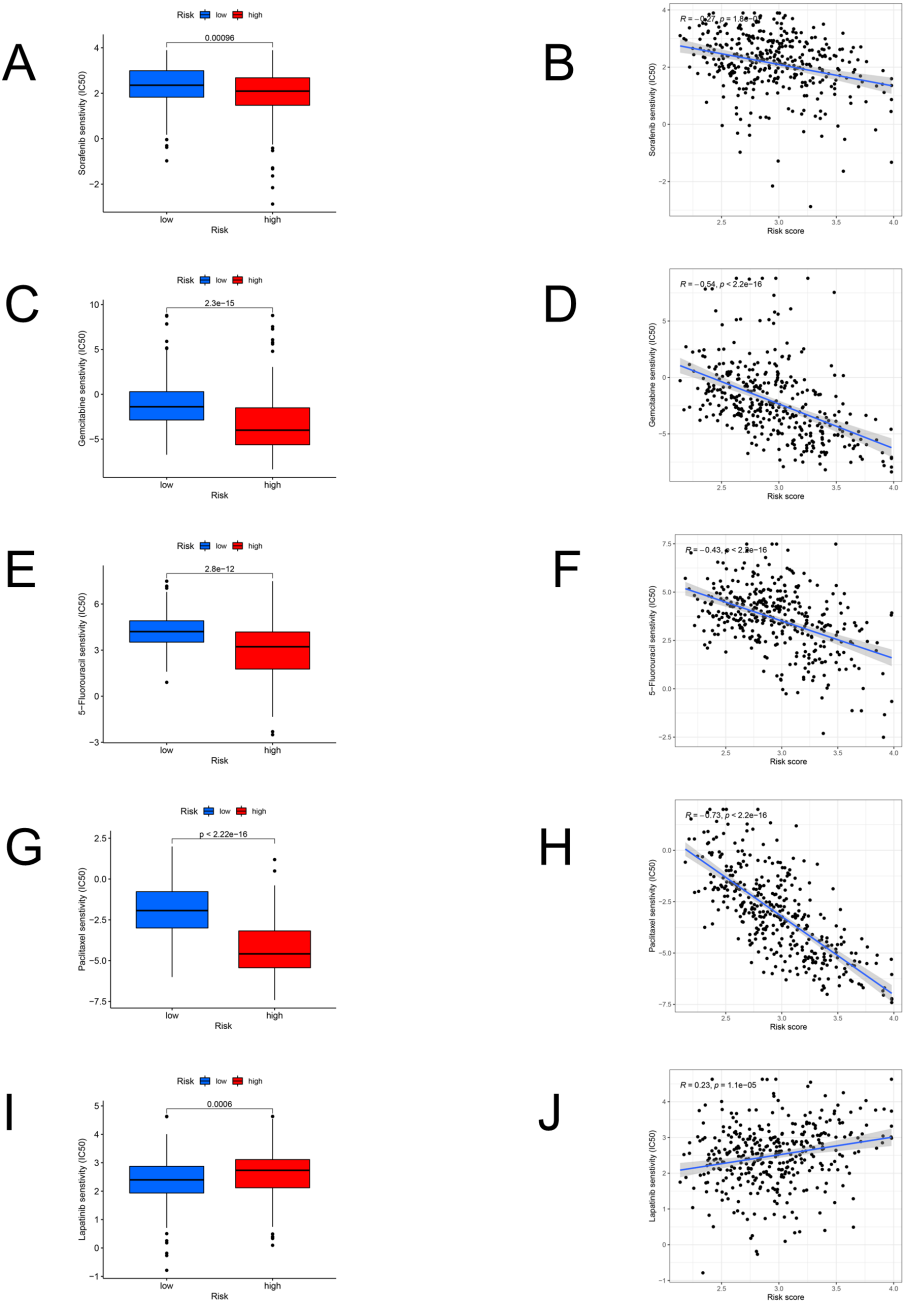
The model in chemotherapy. Relationships between risk scores and chemotherapeutic sensitivity. IC50s of sorafenib (A), gemcitabine (C), 5-fluorouracil (E), paclitaxel (G), and lapatinib (I) in the low- and high-risk groups. The relationship between the risk score and sensitivity to sorafenib (B), gemcitabine (D), 5-fluorouracil (F), paclitaxel (H), and lapatinib (J). IC50: half-maximal inhibitory concentration.

We identified the candidate drugs by the FAM-related risk signature (Fig. 8). Patients from the high-risk set were more sensitive to sorafenib (*P* < 0.001), gemcitabine (*P* < 0.001), 5-fluorouracil (*P* < 0.001), paclitaxel (*P* < 0.001), and the sensitivity to these chemotherapeutic drugs was negatively connected with the risk score. However, lapatinib (*P* < 0.001) was more suitable for the low-risk patients and there was a positive relationship between the sensitivity of chemotherapeutic drugs and risk scores.

### Validating the genes in cells by RT-PCR and western blot

**Figure 9:**
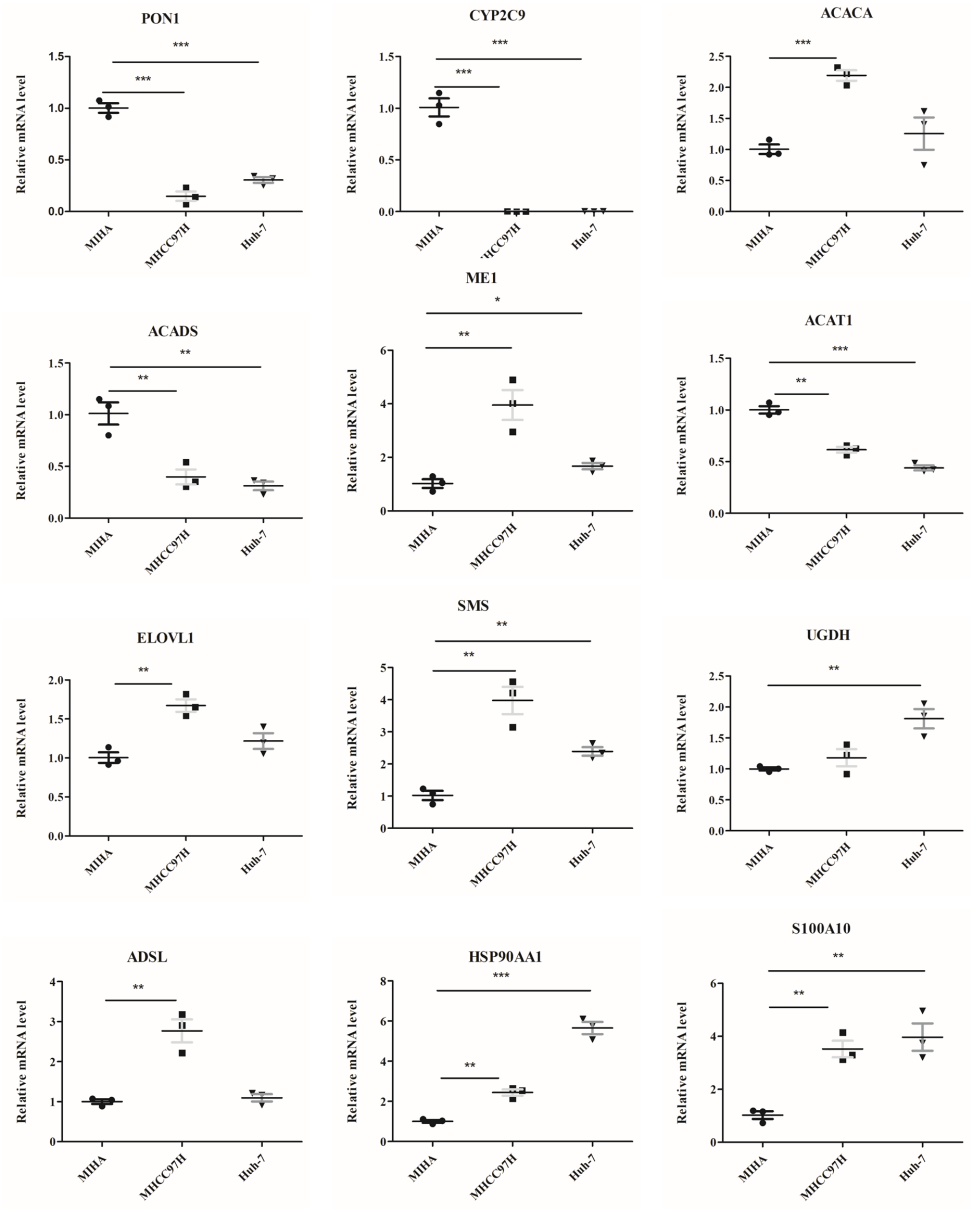
The mRNA expression levels of the related genes. RT-PCR analysis of the 12 model genes in MIHA, MHCC97H, and Huh-7 cells. Data are presented as the mean ± SD, * *P* < 0.05, ** *P* < 0.01 and *** *P* < 0.001.

The mRNA levels of 12 genes between hepatocyte cell line and HCC cell lines were evaluated (Fig. 9). In comparison to MIHA cell line, the expressions of PON1, CYP2C9, ACADS, and ACAT1 were significantly lower in the MHCC97H and Huh-7 cell lines. In addition, the levels of ME1, HSP90AA1, S100A10, ACACA, ELOVL1, SMS, and ADSL were higher in the MHCC97H cell line, and the levels of ME1, HSP90AA1, S100A10, SMS, and UGDH were higher in the Huh-7 cell line compared with MIHA cell line.

**Figure 10:**
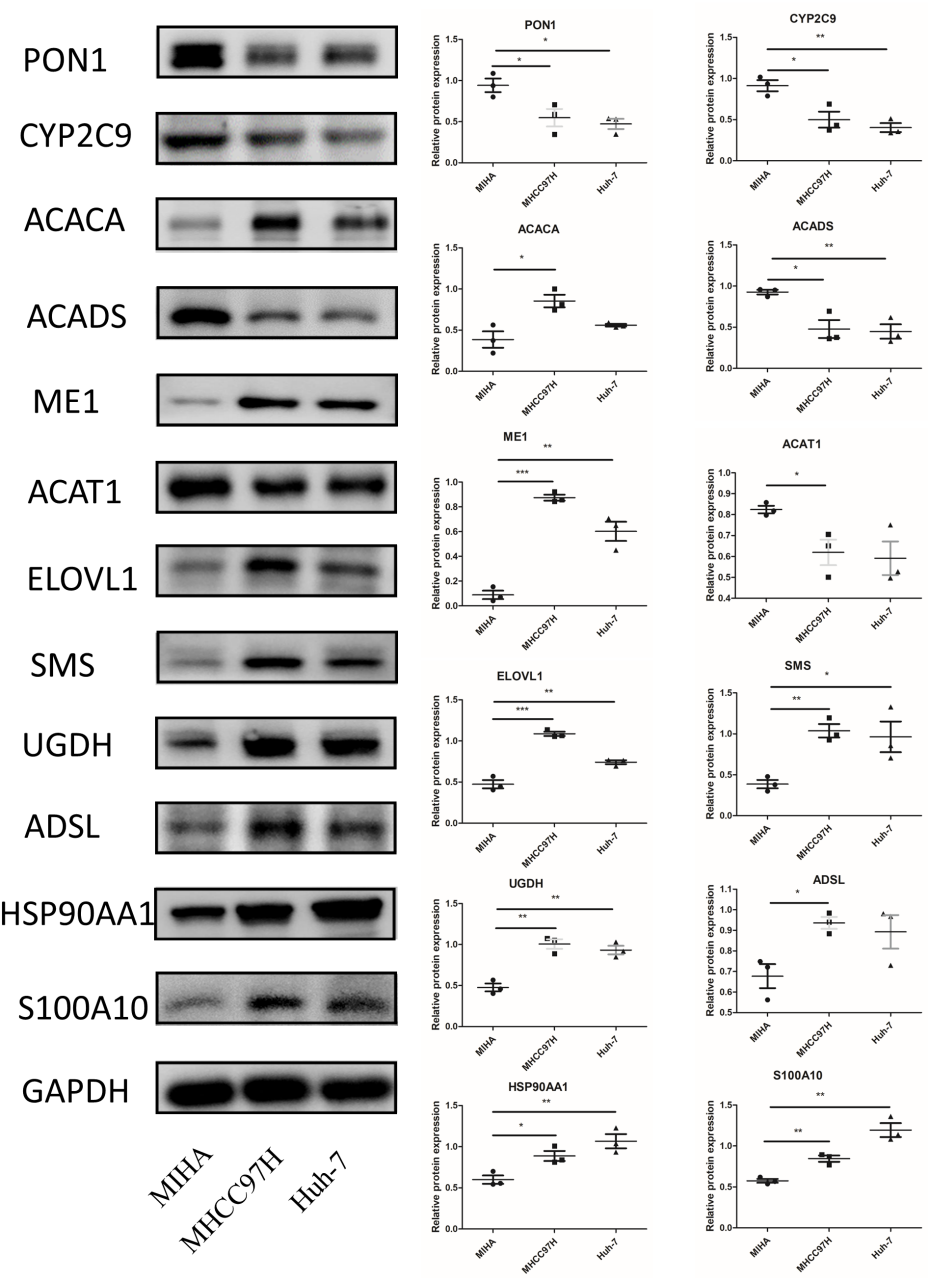
The protein expression of the related genes. Western blot analysis of the 12 model genes in MIHA, MHCC97H, and Huh-7 cells. Data are presented as the mean ± SD, * *P* < 0.05, ** *P* < 0.01, and *** *P* < 0.001.

In Fig. 10, we assessed the protein levels of the risk-score genes in these cell lines by western blot. The levels of ACACA, ME1, ELOVL1, SMS, UGDH, ADSL, HSP90AA1, and S100A10 were significantly higher in MHCC97H cells than in MIHA cells, and the levels of ME1, ELOVL1, SMS, UGDH, HSP90AA1, and S100A10 were higher in Huh-7 cells than in MIHA cells. In addition, the levels of PON1, CYP2C9, ACADS, and ACAT1 were lower in MHCC97H cells than in MIHA cells, and the levels of PON1, CYP2C9, and ACADS were significantly lower in Huh-7 cells than in MIHA cells.

## Discussion

In this study, we analyzed the DEFAMGs between patients with HCC and controls from the TCGA database. Next, the prognosis-related genes were identified and the novel prognostic model for patients with HCC was developed. Besides, we validated the model as an effective biomarker in the GEO database (GSE14520). Furthermore, we demonstrated the risk score model could forecast the OS, identify the response to immunotherapy, select the suitable chemotherapy drugs, and provide the guideline for the clinical work. Notably, we conducted some experimental validations to prove the gene model.

In this study, a total of 12 genes (PON1, CYP2C9, ACACA, ACADS, ME1, ACAT1, ELOVL1, SMS, UGDH, ADSL, HSP90AA1, and S100A10) were used to build the prognostic signature. PON1 is involved in inhibiting the adhesion of leukocytes, reducing the chronic inflammation, and suppressing the tumor invasion or metastasis (Ding et al., 2020). ACADS was reported to participate in the proliferation and metastasis of HCC (Chen et al., 2019a). SMS can influence the metabolic process and is involved in tumor growth (Guo et al., 2020).Overexpression of ACACA, ME1, ADSL or S100A10 promotes HCC growth, migration or invasion, and is connected with poor prognosis (Jiang et al., 2021; Nakashima et al., 2018; Wang et al., 2016b). As for the ACAT1 and CYP2C9, overexpression inhibits the proliferation and migration of tumor cells (Chen et al., 2019b; Yu et al., 2015). Concerning the ELOVL1, UGDH and HSP90AA1, studies only showed the correlation with cancer, and further exploration is needed to elucidate the underlying mechanisms (Fan et al., 2009; Hama et al., 2021; Shi et al., 2020; Yamashita et al., 2017). As in our study, the levels of PON1, CYP2C9, ACADS, and ACAT1 were decreased in the HCC and positively associated with the OS of patients with HCC. The expressions of SMS, HSP90AA1, ADSL, UGDH, ACACA, ME1, ELOVL1, and S100A10 were increased in the tumor tissues and negatively associated with the OS. Notably, our experiments in normal hepatocyte and HCC cell lines confirmed these changes.

In recent years, increasing incidence and mortality of HCC has been a wide concern. However, the prognosis analysis of patients with HCC is mainly being conducted on the basis of the conventional staging, which is not sensitive enough (Bruix, Reig & Sherman, 2016). Therefore, identifying reliable prognostic markers is necessary to improve the clinical outcomes of HCC. Recently, it was reported that abnormal metabolism was strongly connected with the course of tumorigenesis and metastasis (Thakur & Chen, 2019), and the altered FAM in cancer drew the renewed interest (Koundouros & Poulogiannis, 2020). Some studies have revealed that orchestrating fatty acid metabolism can regulate the occurrence and development of HCC (Chen et al., 2021; Ma et al., 2021; Wu et al., 2020). Notably, lots of metabolism-related genes have been revealed to be valuable prognostic markers and metabolism-related risk signatures was built to predict OS of HCC (Hu, Yang & Sang, 2020; Zhao et al., 2021). However, little study on the FAM-related genes risk model has been done. Therefore, we re-screened the FAM-related genes and finally identified 12 genes to construct the prognostic signature. According to the gene model, patients can be divided into high- and low-risk groups. K-M curves revealed that high-risk patients had a shorter survival time than low-risk patients. Furthermore, the predictive capacity of the model was evaluated. The AUCs for 1-, 3-, and 5-year survival were 0.790, 0.685, and 0.698, respectively, which are higher than those in a previous study (Chen, Yang & Chen, 2022) and indicated the accuracy of our model. In addition, univariate and multivariate Cox analyses revealed that the model may be applied as an independent prediction factor in patients with HCC.

Immune system is regarded to play an important role in preventing people from cancer. In recent years, tumor immunotherapy is emerging as a promising adjuvant therapy for HCC (Schoenberg et al., 2021). Tumor immunotherapy aimed at strengthening or weakening the abnormal immune state to control tumor growth or kill tumor cells (Farkona, Diamandis & Blasutig, 2016). However, only part of patients with HCC were reported to be sensitive to the tumor immunotherapy. Wu et al. reported that FAM could adjust the phenotype and function of immune cells, and thus, influence the effect of immunotherapy (Wu et al., 2019). In this study, according to the FAM-related genes model, we found that T cells CD8 was downregulated while the Macrophages M0 was increased in high-risk patients, which suggested the more serious immune disorder in patients with high risk (Zhao et al., 2021; Zhou et al., 2016). Therefore, these patients may be more suitable for immunological therapy. TIDE is regarded as an important immune response biomarker (Jiang et al., 2018). Patients with a low TIDE score are less likely to evade anti-tumor immunity, and immunotherapy is likely to be more effective. In our study, high-risk patients had a lower TIDE score than low-risk patients, suggesting they may respond better to immunotherapy. Furthermore, we explored the reactivity of the model in predicting patients’ response to immunotherapeutic agents. We found that patients with a higher risk score had higher levels of PD-1 and CTLA-4 than those with a lower risk score, which suggested that anti-PD1 and anti-CTLA-4 antibody therapies may be more suitable for high-risk score patients. Collectively, the FAM-related genes model may provide the valuable information to identify HCC patients fit for immunotherapy.

It is reported that different patients respond inconsistently to chemotherapy drugs. Therefore, the individualized treatment should be taken into consideration to improve the clinical effect. Ding et al. (2021) constructed the FAM-related risk signatures to forecast the effect of 5-fluorouracil in colorectal cancer and revealed lower risk patients were more sensitive to 5-fluorouracil. In this study, we demonstrated that patients with HCC with high-risk score were more sensitive to sorafenib, gemcitabine, 5-fluorouracil, and paclitaxel than those with low-risk score; low-risk patients were more sensitive to lapatinib. The different chemotherapeutic sensitivities may be attributed to the different anti-tumor mechanisms of these drugs. Sorafenib is a multi-kinase inhibitor that inhibits vascular endothelial growth factor receptor, thereby reducing angiogenesis and suppressing tumor cell proliferation of HCC (Tang et al., 2020). Gemcitabine belongs to a class of anti-tumor drugs that disrupts cell cycle progression and mainly acts on tumor cells during DNA synthesis, causing DNA breakage and further leading to the tumor cell death of HCC (Kumar et al., 2022). 5-fluorouracil is an anti-metabolic chemotherapeutic drug that can suppress thymidine synthase, thus, inhibiting the synthesis of DNA in hepatocellular tumors (Zhou et al., 2020). Paclitaxel is a naturally occurring anti-tumor agent, which is able to induce cell growth arrest at the G2/M phase, decrease cell proliferation, accelerate apoptosis, and further suppress HCC tumorigenesis (Liu et al., 2020). Lapatinib is a tyrosine kinase inhibitor that can inhibit the epidermal growth factor receptor, and further suppress cell proliferation and tumor growth of HCC (Ding et al., 2017). Collectively, the different anti-tumor mechanisms of these drugs may underlie the different sensitivities between the low- and high-risk patients, and the appropriate chemotherapy can be chosen using the risk score for patients with HCC in the future.

However, this study also has limitation without the clinical trials. Therefore, further exploration should be carried out to evaluate the functions of FAM-related genes in patients with HCC in future.

## Conclusions

In conclusion, we screened the DEFAMGs in patients with HCC using comprehensive bioinformatic analyses. Then, 12 genes related to prognosis were used to build a risk model, which was independently connected with the OS for patients with HCC. This model was externally validated, suggesting its broad applicability. The findings provided a novel, effective and precise prognostic predicting model for patients with HCC. Moreover, the risk model could provide the guidance for personalized immunotherapy and chemotherapy.

##  Supplemental Information

10.7717/peerj.14622/supp-1 Figure S1 Enrichment analysis of DEFAMGs (A-B) Gene ontology enrichment analysis and (C-D) Kyoto Encyclopedia of Genes and Genomes enrichment analysis.

10.7717/peerj.14622/supp-2 Supplemental Information 2 Supplemental Tables

10.7717/peerj.14622/supp-3 Supplemental Information 3 Raw data for RT-PCR analysis of the 12 model genes in MIHA, MHCC97H, and Huh-7 cells

10.7717/peerj.14622/supp-4 Supplemental Information 4 Raw data for western blot analysis of the 12 model genes in MIHA, MHCC97H, and Huh-7 cells

10.7717/peerj.14622/supp-5 Supplemental Information 5 Raw image for western blot

10.7717/peerj.14622/supp-6 File S1 The relevant codes used in this study

## Additional Information and Declarations

### Data Availability

The following information was supplied regarding data availability: The raw measurements are available in the Supplementary Files.
