# Supplementary figures and images for: A novel fatty acid metabolism-related gene prognostic signature and candidate drugs for patients with hepatocellular carcinoma
Source: PeerJ. 2023 Jan 6;11:e14622. doi: 10.7717/peerj.14622 (PMC9828273; doi:10.7717/peerj.14622)

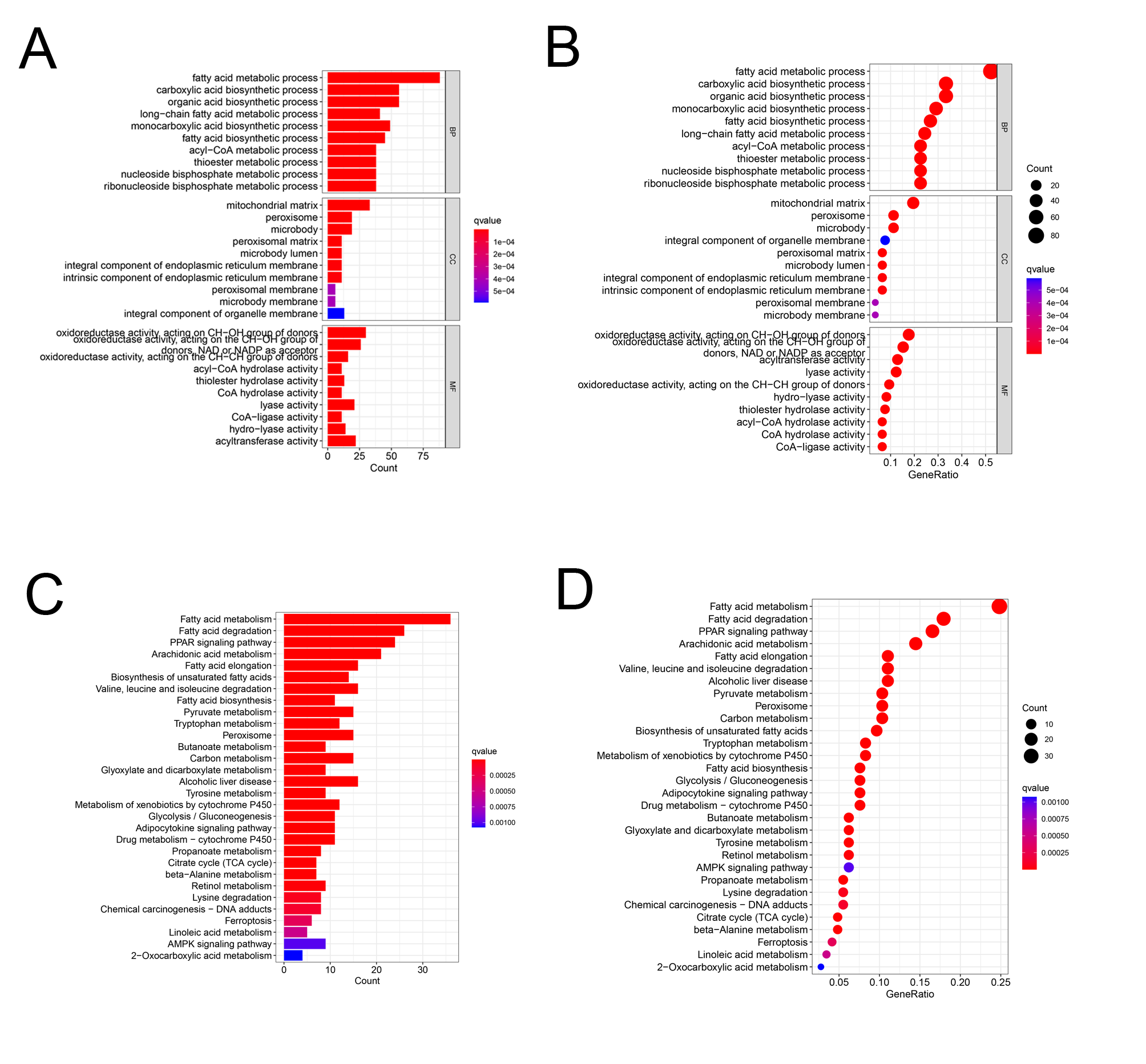

Supplement: Figure S1 — (A-B) Gene ontology enrichment analysis and (C-D) Kyoto Encyclopedia of Genes and Genomes enrichment analysis. [file peerj-11-14622-s001.png]

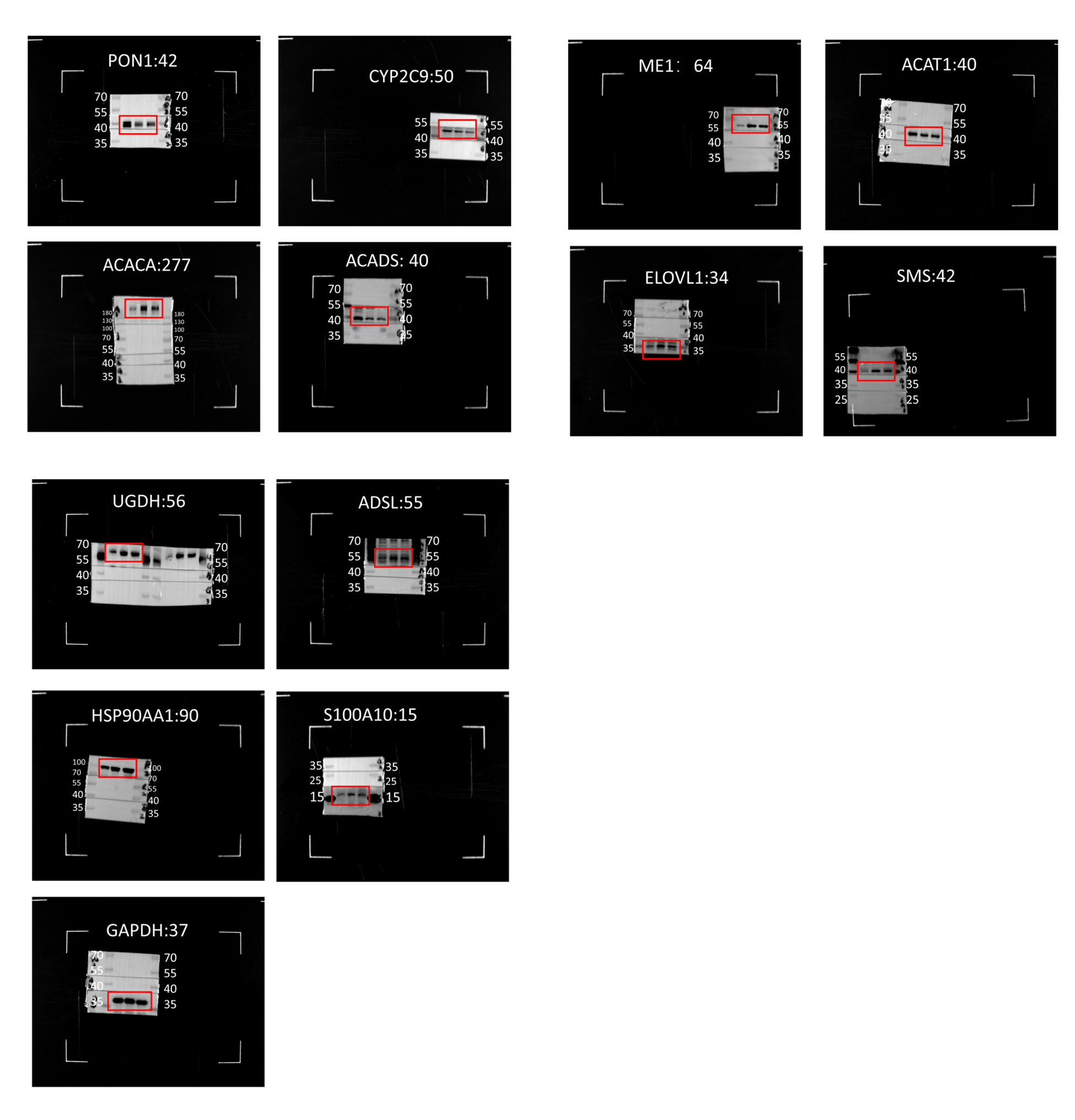

Supplement: Supplemental Information 5 [file peerj-11-14622-s005.png]
