# Supplementary material for: A novel fatty acid metabolism-related gene prognostic signature and candidate drugs for patients with hepatocellular carcinoma
Source: PeerJ. 2023 Jan 6;11:e14622. doi: 10.7717/peerj.14622 (PMC9828273; doi:10.7717/peerj.14622)
Supplement: File S1 [file peerj-11-14622-s006.docx]

Supplementary file of the scripts

1

library(limma)

expFile="symbol.txt"

geneFile="gene.txt"

setwd("D:\\144fatty\\07.FAMexp")

rt=read.table(expFile, header=T, sep="\t", check.names=F)

rt=as.matrix(rt)

rownames(rt)=rt[,1]

exp=rt[,2:ncol(rt)]

dimnames=list(rownames(exp), colnames(exp))

data=matrix(as.numeric(as.matrix(exp)), nrow=nrow(exp), dimnames=dimnames)

data=avereps(data)

data=data[rowMeans(data)>0,]

gene=read.table(geneFile, header=F, sep="\t", check.names=F)

sameGene=intersect(as.vector(gene[,1]), rownames(data))

geneExp=data[sameGene,]

out=rbind(ID=colnames(geneExp),geneExp)

write.table(out,file="FAMexp.txt",sep="\t",quote=F,col.names=F)

2

library(limma)

library(pheatmap)

expFile="FAMexp.txt"

logFCfilter=0.585

fdrFilter=0.05

setwd("D:\\144fatty\\12.diff")

rt=read.table(expFile, header=T, sep="\t", check.names=F)

rt=as.matrix(rt)

rownames(rt)=rt[,1]

exp=rt[,2:ncol(rt)]

dimnames=list(rownames(exp),colnames(exp))

data=matrix(as.numeric(as.matrix(exp)),nrow=nrow(exp),dimnames=dimnames)

data=avereps(data)

data=data[rowMeans(data)>0,]

group=sapply(strsplit(colnames(data),"\\-"),"[",4)

group=sapply(strsplit(group,""),"[",1)

group=gsub("2", "1", group)

conNum=length(group[group==1])

treatNum=length(group[group==0])

grade=c(rep(1,conNum), rep(2,treatNum))

outTab=data.frame()

for(i in row.names(data)){

geneName=unlist(strsplit(i,"\\|",))[1]

geneName=gsub("\\/", "_", geneName)

rt=rbind(expression=data[i,], grade=grade)

rt=as.matrix(t(rt))

wilcoxTest=wilcox.test(expression ~ grade, data=rt)

pvalue=wilcoxTest$p.value

conGeneMeans=mean(data[i,1:conNum])

treatGeneMeans=mean(data[i,(conNum+1):ncol(data)])

logFC=log2(treatGeneMeans)-log2(conGeneMeans)

conMed=median(data[i,1:conNum])

treatMed=median(data[i,(conNum+1):ncol(data)])

diffMed=treatMed-conMed

if( ((logFC>0) & (diffMed>0)) | ((logFC<0) & (diffMed<0)) ){

outTab=rbind(outTab,cbind(gene=i,conMean=conGeneMeans,treatMean=treatGeneMeans,logFC=logFC,pValue=pvalue))

}

}

pValue=outTab[,"pValue"]

fdr=p.adjust(as.numeric(as.vector(pValue)), method="fdr")

outTab=cbind(outTab, fdr=fdr)

write.table(outTab,file="all.txt",sep="\t",row.names=F,quote=F)

outDiff=outTab[( abs(as.numeric(as.vector(outTab$logFC)))>logFCfilter & as.numeric(as.vector(outTab$fdr))<fdrFilter),]

write.table(outDiff,file="diff.txt",sep="\t",row.names=F,quote=F)

heatmap=rbind(ID=colnames(data[as.vector(outDiff[,1]),]),data[as.vector(outDiff[,1]),])

write.table(heatmap,file="diffGeneExp.txt",sep="\t",col.names=F,quote=F)

geneNum=100

outDiff=outDiff[order(as.numeric(as.vector(outDiff$logFC))),]

diffGeneName=as.vector(outDiff[,1])

diffLength=length(diffGeneName)

hmGene=c()

if(diffLength>(2*geneNum)){

hmGene=diffGeneName[c(1:geneNum,(diffLength-geneNum+1):diffLength)]

}else{

hmGene=diffGeneName

}

hmExp=log2(data[hmGene,]+0.01)

Type=c(rep("Normal",conNum),rep("Tumor",treatNum))

names(Type)=colnames(data)

Type=as.data.frame(Type)

pdf(file="heatmap.pdf", width=10, height=7)

pheatmap(hmExp,

annotation=Type,

color = colorRampPalette(c(rep("blue",5), "white", rep("red",5)))(50),

cluster_cols =F,

show_colnames = F,

scale="row",

fontsize = 8,

fontsize_row=5,

fontsize_col=8)

dev.off()

pdf(file="vol.pdf", width=5, height=5)

xMax=6

yMax=max(-log10(outTab$fdr))+1

plot(as.numeric(as.vector(outTab$logFC)), -log10(outTab$fdr), xlab="logFC",ylab="-log10(fdr)",

main="Volcano", ylim=c(0,yMax),xlim=c(-xMax,xMax),yaxs="i",pch=20, cex=1.2)

diffSub=subset(outTab, fdr<fdrFilter & as.numeric(as.vector(logFC))>logFCfilter)

points(as.numeric(as.vector(diffSub$logFC)), -log10(diffSub$fdr), pch=20, col="red",cex=1.5)

diffSub=subset(outTab, fdr<fdrFilter & as.numeric(as.vector(logFC))<(-logFCfilter))

points(as.numeric(as.vector(diffSub$logFC)), -log10(diffSub$fdr), pch=20, col="blue",cex=1.5)

abline(v=0,lty=2,lwd=3)

dev.off()

3.

library(limma)

library(sva)

tcgaExpFile="symbol.txt"

geoExpFile="geoMatrix.txt"

geneFile="diff.txt"

setwd("D:\\144fatty\\13.intersect")

rt=read.table(tcgaExpFile, header=T, sep="\t", check.names=F)

rt=as.matrix(rt)

rownames(rt)=rt[,1]

exp=rt[,2:ncol(rt)]

dimnames=list(rownames(exp),colnames(exp))

tcga=matrix(as.numeric(as.matrix(exp)),nrow=nrow(exp),dimnames=dimnames)

tcga=avereps(tcga)

tcga=log2(tcga+1)

group=sapply(strsplit(colnames(tcga),"\\-"), "[", 4)

group=sapply(strsplit(group,""), "[", 1)

group=gsub("2", "1", group)

tcga=tcga[,group==0]

tcga=t(tcga)

rownames(tcga)=gsub("(.*?)\\-(.*?)\\-(.*?)\\-.*", "\\1\\-\\2\\-\\3", rownames(tcga))

tcga=t(avereps(tcga))

rt=read.table(geoExpFile, header=T, sep="\t", check.names=F)

rt=as.matrix(rt)

rownames(rt)=rt[,1]

exp=rt[,2:ncol(rt)]

dimnames=list(rownames(exp),colnames(exp))

geo=matrix(as.numeric(as.matrix(exp)),nrow=nrow(exp),dimnames=dimnames)

geo=avereps(geo)

qx=as.numeric(quantile(geo, c(0, 0.25, 0.5, 0.75, 0.99, 1.0), na.rm=T))

LogC=( (qx[5]>100) || ( (qx[6]-qx[1])>50 && qx[2]>0) )

if(LogC){

geo[geo<0]=0

geo=log2(geo+1)}

geo=normalizeBetweenArrays(geo)

sameGene=intersect(row.names(tcga),row.names(geo))

tcgaOut=tcga[sameGene,]

geoOut=geo[sameGene,]

all=cbind(tcgaOut,geoOut)

batchType=c(rep(1,ncol(tcgaOut)),rep(2,ncol(geoOut)))

outTab=ComBat(all, batchType, par.prior=TRUE)

tcgaOut=outTab[,colnames(tcgaOut)]

tcgaOut[tcgaOut<0]=0

geoOut=outTab[,colnames(geoOut)]

geoOut[geoOut<0]=0

tcgaTab=rbind(ID=colnames(tcgaOut), tcgaOut)

write.table(tcgaTab, file="TCGA.normalize.txt", sep="\t", quote=F, col.names=F)

geoTab=rbind(ID=colnames(geoOut), geoOut)

write.table(geoTab,file="GEO.normalize.txt",sep="\t",quote=F,col.names=F)

gene=read.table(geneFile, header=T, sep="\t", check.names=F)

sameGene=intersect(as.vector(gene[,1]), rownames(tcgaOut))

tcgaShareExp=tcgaOut[sameGene,]

geoShareExp=geoOut[sameGene,]

tcgaShareExp=rbind(ID=colnames(tcgaShareExp),tcgaShareExp)

write.table(tcgaShareExp,file="TCGA.share.txt",sep="\t",quote=F,col.names=F)

geoShareExp=rbind(ID=colnames(geoShareExp),geoShareExp)

write.table(geoShareExp,file="GEO.share.txt",sep="\t",quote=F,col.names=F)

4

library(limma)

expFile="tcga.share.txt"

cliFile="time.txt"

setwd("D:\\144fatty\\14.tcgaMergeTime")

rt=read.table(expFile, header=T, sep="\t", check.names=F)

rt=as.matrix(rt)

rownames(rt)=rt[,1]

exp=rt[,2:ncol(rt)]

dimnames=list(rownames(exp),colnames(exp))

data=matrix(as.numeric(as.matrix(exp)),nrow=nrow(exp),dimnames=dimnames)

data=avereps(data)

data=data[rowMeans(data)>0,]

data=t(data)

cli=read.table(cliFile, header=T, sep="\t", check.names=F, row.names=1)

sameSample=intersect(row.names(data), row.names(cli))

data=data[sameSample,]

cli=cli[sameSample,]

out=cbind(cli, data)

out=cbind(id=row.names(out), out)

write.table(out, file="TCGA.expTime.txt", sep="\t", row.names=F, quote=F)

5

library(limma)

expFile="geo.share.txt"

cliFile="time.txt"

setwd("D:\\144fatty\\15.geoMergeTime")

rt=read.table(expFile, header=T, sep="\t", check.names=F)

rt=as.matrix(rt)

rownames(rt)=rt[,1]

exp=rt[,2:ncol(rt)]

dimnames=list(rownames(exp),colnames(exp))

data=matrix(as.numeric(as.matrix(exp)),nrow=nrow(exp),dimnames=dimnames)

data=avereps(data)

data=data[rowMeans(data)>0,]

data=t(data)

cli=read.table(cliFile, header=T, sep="\t", check.names=F, row.names=1)

sameSample=intersect(row.names(data),row.names(cli))

data=data[sameSample,]

cli=cli[sameSample,]

out=cbind(cli,data)

out=cbind(id=row.names(out),out)

write.table(out,file="GEO.expTime.txt",sep="\t",row.names=F,quote=F)

6

library(survival)

library(survminer)

coxPfilter=0.01

inputFile="TCGA.expTime.txt"

setwd("D:\\144fatty\\16.uniCox")

rt=read.table(inputFile, header=T, sep="\t", check.names=F, row.names=1)

rt$futime=rt$futime/365

outTab=data.frame()

sigGenes=c("futime","fustat")

for(i in colnames(rt[,3:ncol(rt)])){

cox <- coxph(Surv(futime, fustat) ~ rt[,i], data = rt)

coxSummary = summary(cox)

coxP=coxSummary$coefficients[,"Pr(>|z|)"]

if(coxP<coxPfilter){

sigGenes=c(sigGenes,i)

outTab=rbind(outTab,

cbind(id=i,

HR=coxSummary$conf.int[,"exp(coef)"],

HR.95L=coxSummary$conf.int[,"lower .95"],

HR.95H=coxSummary$conf.int[,"upper .95"],

pvalue=coxSummary$coefficients[,"Pr(>|z|)"])

)

}

}

write.table(outTab,file="TCGA.uniCox.txt",sep="\t",row.names=F,quote=F)

uniSigExp=rt[,sigGenes]

uniSigExp=cbind(id=row.names(uniSigExp),uniSigExp)

write.table(uniSigExp,file="TCGA.uniSigExp.txt",sep="\t",row.names=F,quote=F)

bioForest=function(coxFile=null,forestFile=null,forestCol=null){

rt <- read.table(coxFile,header=T,sep="\t",row.names=1,check.names=F)

gene <- rownames(rt)

hr <- sprintf("%.3f",rt$"HR")

hrLow <- sprintf("%.3f",rt$"HR.95L")

hrHigh <- sprintf("%.3f",rt$"HR.95H")

Hazard.ratio <- paste0(hr,"(",hrLow,"-",hrHigh,")")

pVal <- ifelse(rt$pvalue<0.001, "<0.001", sprintf("%.3f", rt$pvalue))

height=nrow(rt)/12.5+5

pdf(file=forestFile, width = 7,height = height)

n <- nrow(rt)

nRow <- n+1

ylim <- c(1,nRow)

layout(matrix(c(1,2),nc=2),width=c(3,2.5))

xlim = c(0,3)

par(mar=c(4,2.5,2,1))

plot(1,xlim=xlim,ylim=ylim,type="n",axes=F,xlab="",ylab="")

text.cex=0.8

text(0,n:1,gene,adj=0,cex=text.cex)

text(1.5-0.5*0.2,n:1,pVal,adj=1,cex=text.cex);text(1.5-0.5*0.2,n+1,'pvalue',cex=text.cex,font=2,adj=1)

text(3,n:1,Hazard.ratio,adj=1,cex=text.cex);text(3,n+1,'Hazard ratio',cex=text.cex,font=2,adj=1,)

par(mar=c(4,1,2,1),mgp=c(2,0.5,0))

xlim = c(0,max(as.numeric(hrLow),as.numeric(hrHigh)))

plot(1,xlim=xlim,ylim=ylim,type="n",axes=F,ylab="",xaxs="i",xlab="Hazard ratio")

arrows(as.numeric(hrLow),n:1,as.numeric(hrHigh),n:1,angle=90,code=3,length=0.05,col="darkblue",lwd=2.5)

abline(v=1,col="black",lty=2,lwd=2)

boxcolor = ifelse(as.numeric(hr) > 1, forestCol[1], forestCol[2])

points(as.numeric(hr), n:1, pch = 15, col = boxcolor, cex=1.6)

axis(1)

dev.off()

}

bioForest(coxFile="TCGA.uniCox.txt",forestFile="forest.pdf",forestCol=c("red","blue"))

7

library("glmnet")

library("survival")

set.seed(12345)

trainFile="TCGA.uniSigExp.txt"

testFile="GEO.expTime.txt"

setwd("D:\\144fatty\\18.model")

rt=read.table(trainFile, header=T, sep="\t", row.names=1)

rt$futime[rt$futime<=0]=0.003

x=as.matrix(rt[,c(3:ncol(rt))])

y=data.matrix(Surv(rt$futime,rt$fustat))

fit=glmnet(x, y, family = "cox", maxit = 1000)

pdf("lasso.lambda.pdf")

plot(fit, xvar="lambda", label=TRUE)

dev.off()

cvfit=cv.glmnet(x, y, family="cox", maxit=10)

pdf("lasso.cvfit.pdf")

plot(cvfit)

abline(v=log(c(cvfit$lambda.min,cvfit$lambda.1se)), lty="dashed")

dev.off()

coef=coef(fit, s = cvfit$lambda.min)

index=which(coef != 0)

actCoef=coef[index]

lassoGene=row.names(coef)[index]

geneCoef=cbind(Gene=lassoGene,Coef=actCoef)

write.table(geneCoef,file="geneCoef.txt",sep="\t",quote=F,row.names=F)

trainFinalGeneExp=rt[,lassoGene]

myFun=function(x){crossprod(as.numeric(x),actCoef)}

trainScore=apply(trainFinalGeneExp,1,myFun)

outCol=c("futime","fustat",lassoGene)

Risk=as.vector(ifelse(trainScore>median(trainScore),"high","low"))

outTab=cbind(rt[,outCol],riskScore=as.vector(trainScore),Risk)

write.table(cbind(id=rownames(outTab),outTab),file="risk.TCGA.txt",sep="\t",quote=F,row.names=F)

rt=read.table(testFile, header=T, sep="\t", row.names=1)

rt$futime=rt$futime/365

testFinalGeneExp=rt[,lassoGene]

testScore=apply(testFinalGeneExp,1,myFun)

outCol=c("futime","fustat",lassoGene)

Risk=as.vector(ifelse(testScore>median(trainScore),"high","low"))

outTab=cbind(rt[,outCol],riskScore=as.vector(testScore),Risk)

write.table(cbind(id=rownames(outTab),outTab),file="risk.GEO.txt",sep="\t",quote=F,row.names=F)

8

library(limma)

library(ggplot2)

expFile="FAMexp.txt"

riskFile="risk.TCGA.txt"

setwd("D:\\144fatty\\19.PCA")

risk=read.table(riskFile, header=T, sep="\t", check.names=F, row.names=1)

data=risk[,3:(ncol(risk)-2)]

Risk=as.vector(risk[,"Risk"])

data.pca=prcomp(data, scale. = TRUE)

pcaPredict=predict(data.pca)

PCA = data.frame(PC1=pcaPredict[,1], PC2=pcaPredict[,2], Risk)

pdf(file="PCA.modelGene.pdf", width=5.5, height=4.5)

p=ggplot(data = PCA, aes(PC1, PC2)) + geom_point(aes(color = Risk)) +

scale_colour_manual(name="Risk", values =c("red", "blue"))+

theme_bw()+

theme(plot.margin=unit(rep(1.5,4),'lines'))+

theme(panel.grid.major = element_blank(), panel.grid.minor = element_blank())

print(p)

dev.off()

rt=read.table(expFile, header=T, sep="\t", check.names=F)

rt=as.matrix(rt)

rownames(rt)=rt[,1]

exp=rt[,2:ncol(rt)]

dimnames=list(rownames(exp),colnames(exp))

data=matrix(as.numeric(as.matrix(exp)),nrow=nrow(exp),dimnames=dimnames)

data=avereps(data)

data=data[rowMeans(data)>0.5,]

type=sapply(strsplit(colnames(data),"\\-"),"[",4)

type=sapply(strsplit(type,""),"[",1)

type=gsub("2","1",type)

data=t(data[,type==0])

rownames(data)=gsub("(.*?)\\-(.*?)\\-(.*?)\\-(.*?)\\-.*","\\1\\-\\2\\-\\3",rownames(data))

sameSample=intersect(rownames(data),rownames(risk))

data=data[sameSample,]

Risk=risk[sameSample,"Risk"]

data.pca=prcomp(data, scale. = TRUE)

pcaPredict=predict(data.pca)

PCA = data.frame(PC1=pcaPredict[,1], PC2=pcaPredict[,2], Risk)

pdf(file="PCA.FAMgene.pdf", width=5.5, height=4.5)

p=ggplot(data = PCA, aes(PC1, PC2)) + geom_point(aes(color = Risk)) +

scale_colour_manual(name="Risk", values =c("red", "blue"))+

theme_bw()+

theme(plot.margin=unit(rep(1.5,4),'lines'))+

theme(panel.grid.major = element_blank(), panel.grid.minor = element_blank())

print(p)

dev.off()

9

library(survival)

library(survminer)

setwd("D:\\144fatty\\20.survival")

bioSurvival=function(inputFile=null, outFile=null){

rt=read.table(inputFile, header=T, sep="\t", check.names=F)

diff=survdiff(Surv(futime, fustat) ~ Risk,data = rt)

pValue=1-pchisq(diff$chisq,df=1)

if(pValue<0.001){

pValue="p<0.001"

}else{

pValue=paste0("p=",sprintf("%.03f",pValue))

}

fit <- survfit(Surv(futime, fustat) ~ Risk, data = rt)

surPlot=ggsurvplot(fit,

data=rt,

conf.int=T,

pval=pValue,

pval.size=6,

legend.title="Risk",

legend.labs=c("High risk", "Low risk"),

xlab="Time(years)",

ylab="Overall survival",

break.time.by = 1,

palette=c("red", "blue"),

risk.table=TRUE,

risk.table.title="",

risk.table.height=.25)

pdf(file=outFile, width=6.5, height=5.5, onefile=FALSE)

print(surPlot)

dev.off()

}

bioSurvival(inputFile="risk.TCGA.txt", outFile="survival.TCGA.pdf")

bioSurvival(inputFile="risk.GEO.txt", outFile="survival.GEO.pdf")

10

library(survival)

library(survminer)

riskFile="risk.TCGA.txt"

cliFile="Survival_SupplementalTable_S1_20171025_xena_sp"

setwd("D:\\144fatty\\21.PFS")

risk=read.table(riskFile, header=T, sep="\t", check.names=F, row.names=1)

cli=read.table(cliFile, header=T, sep="\t", check.names=F, row.names=1)

cli=cli[,c("PFI.time", "PFI")]

cli=na.omit(cli)

colnames(cli)=c("futime", "fustat")

cli$futime=cli$futime/365

cli=as.matrix(cli)

row.names(cli)=gsub("(.*?)\\-(.*?)\\-(.*?)\\-.*", "\\1\\-\\2\\-\\3", row.names(cli))

sameSample=intersect(row.names(risk), row.names(cli))

rt=cbind(cli[sameSample,,drop=F], risk[sameSample,"Risk",drop=F])

length=length(levels(factor(rt$Risk)))

diff=survdiff(Surv(futime, fustat) ~ Risk, data = rt)

pValue=1-pchisq(diff$chisq, df=length-1)

if(pValue<0.001){

pValue="p<0.001"

}else{

pValue=paste0("p=",sprintf("%.03f",pValue))

}

fit=survfit(Surv(futime, fustat) ~ Risk, data = rt)

#print(surv_median(fit))

surPlot=ggsurvplot(fit,

data=rt,

conf.int=T,

pval=pValue,

pval.size=6,

legend.title="Risk",

legend.labs=c("High risk", "Low risk"),

font.legend=10,

xlab="Time(years)",

ylab="Progression free survival",

break.time.by = 1,

palette = c("red", "blue"),

#surv.median.line = "hv",

risk.table=T,

cumevents=F,

risk.table.height=.25)

pdf(file="PFS.pdf", width=6.5, height=5.5, onefile=FALSE)

print(surPlot)

dev.off()

11

library(survival)

setwd("D:\\144fatty\\22.indep")

bioForest=function(coxFile=null, forestFile=null, forestCol=null){

rt <- read.table(coxFile, header=T, sep="\t", check.names=F, row.names=1)

gene <- rownames(rt)

hr <- sprintf("%.3f",rt$"HR")

hrLow <- sprintf("%.3f",rt$"HR.95L")

hrHigh <- sprintf("%.3f",rt$"HR.95H")

Hazard.ratio <- paste0(hr,"(",hrLow,"-",hrHigh,")")

pVal <- ifelse(rt$pvalue<0.001, "<0.001", sprintf("%.3f", rt$pvalue))

pdf(file=forestFile, width=6.5, height=4.5)

n <- nrow(rt)

nRow <- n+1

ylim <- c(1,nRow)

layout(matrix(c(1,2),nc=2),width=c(3,2.5))

xlim = c(0,3)

par(mar=c(4,2.5,2,1))

plot(1,xlim=xlim,ylim=ylim,type="n",axes=F,xlab="",ylab="")

text.cex=0.8

text(0,n:1,gene,adj=0,cex=text.cex)

text(1.5-0.5*0.2,n:1,pVal,adj=1,cex=text.cex);text(1.5-0.5*0.2,n+1,'pvalue',cex=text.cex,font=2,adj=1)

text(3.1,n:1,Hazard.ratio,adj=1,cex=text.cex);text(3.1,n+1,'Hazard ratio',cex=text.cex,font=2,adj=1)

par(mar=c(4,1,2,1),mgp=c(2,0.5,0))

xlim = c(0,max(as.numeric(hrLow),as.numeric(hrHigh)))

plot(1,xlim=xlim,ylim=ylim,type="n",axes=F,ylab="",xaxs="i",xlab="Hazard ratio")

arrows(as.numeric(hrLow),n:1,as.numeric(hrHigh),n:1,angle=90,code=3,length=0.05,col="darkblue",lwd=3)

abline(v=1, col="black", lty=2, lwd=2)

boxcolor = ifelse(as.numeric(hr) > 1, forestCol, forestCol)

points(as.numeric(hr), n:1, pch = 15, col = boxcolor, cex=2)

axis(1)

dev.off()

}

indep=function(riskFile=null,cliFile=null,uniOutFile=null,multiOutFile=null,uniForest=null,multiForest=null){

risk=read.table(riskFile, header=T, sep="\t", check.names=F, row.names=1)

cli=read.table(cliFile, header=T, sep="\t", check.names=F, row.names=1)

sameSample=intersect(row.names(cli),row.names(risk))

risk=risk[sameSample,]

cli=cli[sameSample,]

rt=cbind(futime=risk[,1], fustat=risk[,2], cli, riskScore=risk[,(ncol(risk)-1)])

uniTab=data.frame()

for(i in colnames(rt[,3:ncol(rt)])){

cox <- coxph(Surv(futime, fustat) ~ rt[,i], data = rt)

coxSummary = summary(cox)

uniTab=rbind(uniTab,

cbind(id=i,

HR=coxSummary$conf.int[,"exp(coef)"],

HR.95L=coxSummary$conf.int[,"lower .95"],

HR.95H=coxSummary$conf.int[,"upper .95"],

pvalue=coxSummary$coefficients[,"Pr(>|z|)"])

)

}

write.table(uniTab,file=uniOutFile,sep="\t",row.names=F,quote=F)

bioForest(coxFile=uniOutFile, forestFile=uniForest, forestCol="blue")

uniTab=uniTab[as.numeric(uniTab[,"pvalue"])<1,]

rt1=rt[,c("futime", "fustat", as.vector(uniTab[,"id"]))]

multiCox=coxph(Surv(futime, fustat) ~ ., data = rt1)

multiCoxSum=summary(multiCox)

multiTab=data.frame()

multiTab=cbind(

HR=multiCoxSum$conf.int[,"exp(coef)"],

HR.95L=multiCoxSum$conf.int[,"lower .95"],

HR.95H=multiCoxSum$conf.int[,"upper .95"],

pvalue=multiCoxSum$coefficients[,"Pr(>|z|)"])

multiTab=cbind(id=row.names(multiTab),multiTab)

write.table(multiTab,file=multiOutFile,sep="\t",row.names=F,quote=F)

bioForest(coxFile=multiOutFile, forestFile=multiForest, forestCol="red")

}

indep(riskFile="risk.TCGA.txt",

cliFile="clinical.txt",

uniOutFile="uniCox.txt",

multiOutFile="multiCox.txt",

uniForest="uniForest.pdf",

multiForest="multiForest.pdf")

12

library(survival)

library(survminer)

library(timeROC)

riskFile="risk.TCGA.txt"

cliFile="clinical.txt"

setwd("D:\\144fatty\\23.ROC")

risk=read.table(riskFile, header=T, sep="\t", check.names=F, row.names=1)

risk=risk[,c("futime", "fustat", "riskScore")]

cli=read.table(cliFile, header=T, sep="\t", check.names=F, row.names=1)

samSample=intersect(row.names(risk), row.names(cli))

risk1=risk[samSample,,drop=F]

cli=cli[samSample,,drop=F]

rt=cbind(risk1, cli)

bioCol=rainbow(ncol(rt)-1, s=0.9, v=0.9)

ROC_rt=timeROC(T=risk$futime, delta=risk$fustat,

marker=risk$riskScore, cause=1,

weighting='aalen',

times=c(1,3,5), ROC=TRUE)

pdf(file="ROC.pdf", width=5.5, height=5.5)

plot(ROC_rt,time=1,col=bioCol[1],title=FALSE,lwd=2)

plot(ROC_rt,time=3,col=bioCol[2],add=TRUE,title=FALSE,lwd=2)

plot(ROC_rt,time=5,col=bioCol[3],add=TRUE,title=FALSE,lwd=2)

legend('bottomright',

c(paste0('AUC at 1 years: ',sprintf("%.03f",ROC_rt$AUC[1])),

paste0('AUC at 3 years: ',sprintf("%.03f",ROC_rt$AUC[2])),

paste0('AUC at 5 years: ',sprintf("%.03f",ROC_rt$AUC[3]))),

col=bioCol[1:3], lwd=2, bty = 'n')

dev.off()

predictTime=5

aucText=c()

pdf(file="cliROC.pdf", width=5.5, height=5.5)

i=3

ROC_rt=timeROC(T=risk$futime,

delta=risk$fustat,

marker=risk$riskScore, cause=1,

weighting='aalen',

times=c(predictTime),ROC=TRUE)

plot(ROC_rt, time=predictTime, col=bioCol[i-2], title=FALSE, lwd=2)

aucText=c(paste0("Risk", ", AUC=", sprintf("%.3f",ROC_rt$AUC[2])))

abline(0,1)

for(i in 4:ncol(rt)){

ROC_rt=timeROC(T=rt$futime,

delta=rt$fustat,

marker=rt[,i], cause=1,

weighting='aalen',

times=c(predictTime),ROC=TRUE)

plot(ROC_rt, time=predictTime, col=bioCol[i-2], title=FALSE, lwd=2, add=TRUE)

aucText=c(aucText, paste0(colnames(rt)[i],", AUC=",sprintf("%.3f",ROC_rt$AUC[2])))

}

legend("bottomright", aucText,lwd=2,bty="n",col=bioCol[1:(ncol(rt)-1)])

dev.off()

13

library(survival)

library(survminer)

riskFile="risk.all.txt"

cliFile="clinical.txt"

setwd("D:\\144fatty\\24.cliCor")

risk=read.table(riskFile, header=T, sep="\t", check.names=F, row.names=1)

cli=read.table(cliFile, header=T, sep="\t", check.names=F, row.names=1)

cliName=colnames(cli)[1]

sameSample=intersect(row.names(cli), row.names(risk))

risk=risk[sameSample,,drop=F]

cli=cli[sameSample,,drop=F]

rt=cbind(futime=risk[,1], fustat=risk[,2], cli, risk[,"risk",drop=F])

colnames(rt)=c("futime", "fustat", "clinical", "Risk")

tab=table(rt[,"clinical"])

tab=tab[tab!=0]

for(j in names(tab)){

rt1=rt[(rt[,"clinical"]==j),]

tab1=table(rt1[,"Risk"])

tab1=tab1[tab1!=0]

labels=names(tab1)

if(length(labels)!=2){next}

if((cliName=="age") | (cliName=="Age") | (cliName=="AGE")){

titleName=paste0("age",j)

}

diff=survdiff(Surv(futime, fustat) ~Risk,data = rt1)

pValue=1-pchisq(diff$chisq,df=1)

if(pValue<0.001){

pValue="p<0.001"

}else{

pValue=paste0("p=",sprintf("%.03f",pValue))

}

fit <- survfit(Surv(futime, fustat) ~ Risk, data = rt1)

surPlot=ggsurvplot(fit,

data=rt1,

conf.int=F,

pval=pValue,

pval.size=6,

title=paste0("Patients with ",j),

legend.title="Risk",

legend.labs=labels,

font.legend=12,

xlab="Time(years)",

break.time.by = 1,

palette=c("red", "blue"),

risk.table=F,

risk.table.title="",

risk.table.col = "strata",

risk.table.height=.25)

j=gsub(">=","ge",j);j=gsub("<=","le",j);j=gsub(">","gt",j);j=gsub("<","lt",j)

pdf(file=paste0("survival.",cliName,"_",j,".pdf"), onefile = FALSE,

width = 6,

height =5)

print(surPlot)

dev.off()

}

14

library(survival)

library(regplot)

library(rms)

riskFile="risk.TCGA.txt"

cliFile="clinical.txt"

setwd("D:\\144fatty\\26.Nomo")

risk=read.table(riskFile, header=T, sep="\t", check.names=F, row.names=1)

cli=read.table(cliFile, header=T, sep="\t", check.names=F, row.names=1)

cli=cli[apply(cli,1,function(x)any(is.na(match('unknow',x)))),,drop=F]

cli$Age=as.numeric(cli$Age)

samSample=intersect(row.names(risk), row.names(cli))

risk1=risk[samSample,,drop=F]

cli=cli[samSample,,drop=F]

rt=cbind(risk1[,c("futime", "fustat", "Risk")], cli)

res.cox=coxph(Surv(futime, fustat) ~ . , data = rt)

nom1=regplot(res.cox,

plots = c("density", "boxes"),

clickable=F,

title="",

points=TRUE,

droplines=TRUE,

observation=rt[9,],

rank="sd",

failtime = c(1,3,5),

prfail = F)

nomoRisk=predict(res.cox, data=rt, type="risk")

rt=cbind(risk1, Nomogram=nomoRisk)

outTab=rbind(ID=colnames(rt), rt)

write.table(outTab, file="nomoRisk.txt", sep="\t", col.names=F, quote=F)

pdf(file="calibration.pdf", width=5, height=5)

f <- cph(Surv(futime, fustat) ~ Nomogram, x=T, y=T, surv=T, data=rt, time.inc=1)

cal <- calibrate(f, cmethod="KM", method="boot", u=1, m=(nrow(rt)/3), B=1000)

plot(cal, xlim=c(0,1), ylim=c(0,1),

xlab="Nomogram-predicted OS (%)", ylab="Observed OS (%)", lwd=1.5, col="blue", sub=F)

f <- cph(Surv(futime, fustat) ~ Nomogram, x=T, y=T, surv=T, data=rt, time.inc=3)

cal <- calibrate(f, cmethod="KM", method="boot", u=3, m=(nrow(rt)/3), B=1000)

plot(cal, xlim=c(0,1), ylim=c(0,1), xlab="", ylab="", lwd=1.5, col="blue", sub=F, add=T)

f <- cph(Surv(futime, fustat) ~ Nomogram, x=T, y=T, surv=T, data=rt, time.inc=5)

cal <- calibrate(f, cmethod="KM", method="boot", u=5, m=(nrow(rt)/3), B=1000)

plot(cal, xlim=c(0,1), ylim=c(0,1), xlab="", ylab="", lwd=1.5, col="red", sub=F, add=T)

legend('bottomright', c('1-year', '3-year', '5-year'),

col=c("green","blue","red"), lwd=1.5, bty = 'n')

dev.off()

15

library(survival)

library(survminer)

library(timeROC)

predictTime=5

riskFile="nomoRisk.txt"

cliFile="clinical.txt"

setwd("D:\\144fatty\\27.nomoROC\\HCCXSTAGE")

risk=read.table(riskFile, header=T, sep="\t", check.names=F, row.names=1)

cli=read.table(cliFile, header=T, sep="\t", check.names=F, row.names=1)

samSample=intersect(row.names(risk), row.names(cli))

risk1=risk[samSample,,drop=F]

cli1=cli[samSample,,drop=F]

data=cbind(risk1, cli1)

rt=cbind(risk1[,c("futime","fustat","riskScore","Nomogram")], cli1)

aucText=c()

bioCol=rainbow(ncol(rt)-1, s=0.9, v=0.9)

pdf(file="cliROC.pdf", width=6, height=6)

i=3

ROC_rt=timeROC(T=risk$futime,

delta=risk$fustat,

marker=risk$riskScore, cause=1,

weighting='aalen',

times=c(predictTime),ROC=TRUE)

plot(ROC_rt, time=predictTime, col=bioCol[i-2], title=FALSE, lwd=2)

aucText=c(paste0("Risk", ", AUC=", sprintf("%.3f",ROC_rt$AUC[2])))

abline(0,1)

for(i in 4:ncol(rt)){

ROC_rt=timeROC(T=rt$futime,

delta=rt$fustat,

marker=rt[,i], cause=1,

weighting='aalen',

times=c(predictTime),ROC=TRUE)

plot(ROC_rt, time=predictTime, col=bioCol[i-2], title=FALSE, lwd=2, add=TRUE)

aucText=c(aucText, paste0(colnames(rt)[i],", AUC=",sprintf("%.3f",ROC_rt$AUC[2])))

}

legend("bottomright", aucText,lwd=2,bty="n",col=bioCol[1:(ncol(rt)-1)])

dev.off()

16

library(survival)

setwd("D:\\144fatty\\28.nomoIndep")

bioForest=function(coxFile=null, forestFile=null, forestCol=null){

rt <- read.table(coxFile, header=T, sep="\t", check.names=F, row.names=1)

gene <- rownames(rt)

hr <- sprintf("%.3f",rt$"HR")

hrLow <- sprintf("%.3f",rt$"HR.95L")

hrHigh <- sprintf("%.3f",rt$"HR.95H")

Hazard.ratio <- paste0(hr,"(",hrLow,"-",hrHigh,")")

pVal <- ifelse(rt$pvalue<0.001, "<0.001", sprintf("%.3f", rt$pvalue))

pdf(file=forestFile, width=6.5, height=4.5)

n <- nrow(rt)

nRow <- n+1

ylim <- c(1,nRow)

layout(matrix(c(1,2),nc=2),width=c(3,2.5))

xlim = c(0,3)

par(mar=c(4,2.5,2,1))

plot(1,xlim=xlim,ylim=ylim,type="n",axes=F,xlab="",ylab="")

text.cex=0.8

text(0,n:1,gene,adj=0,cex=text.cex)

text(1.5-0.5*0.2,n:1,pVal,adj=1,cex=text.cex);text(1.5-0.5*0.2,n+1,'pvalue',cex=text.cex,font=2,adj=1)

text(3.1,n:1,Hazard.ratio,adj=1,cex=text.cex);text(3.1,n+1,'Hazard ratio',cex=text.cex,font=2,adj=1)

par(mar=c(4,1,2,1),mgp=c(2,0.5,0))

xlim = c(0,max(as.numeric(hrLow),as.numeric(hrHigh)))

plot(1,xlim=xlim,ylim=ylim,type="n",axes=F,ylab="",xaxs="i",xlab="Hazard ratio")

arrows(as.numeric(hrLow),n:1,as.numeric(hrHigh),n:1,angle=90,code=3,length=0.05,col="darkblue",lwd=3)

abline(v=1, col="black", lty=2, lwd=2)

boxcolor = ifelse(as.numeric(hr) > 1, forestCol, forestCol)

points(as.numeric(hr), n:1, pch = 15, col = boxcolor, cex=2)

axis(1)

dev.off()

}

indep=function(riskFile=null,cliFile=null,uniOutFile=null,multiOutFile=null,uniForest=null,multiForest=null){

risk=read.table(riskFile, header=T, sep="\t", check.names=F, row.names=1)

cli=read.table(cliFile, header=T, sep="\t", check.names=F, row.names=1)

sameSample=intersect(row.names(cli),row.names(risk))

risk=risk[sameSample,]

cli=cli[sameSample,]

rt=cbind(futime=risk[,1], fustat=risk[,2], cli, Nomogram=risk[,ncol(risk)])

uniTab=data.frame()

for(i in colnames(rt[,3:ncol(rt)])){

cox <- coxph(Surv(futime, fustat) ~ rt[,i], data = rt)

coxSummary = summary(cox)

uniTab=rbind(uniTab,

cbind(id=i,

HR=coxSummary$conf.int[,"exp(coef)"],

HR.95L=coxSummary$conf.int[,"lower .95"],

HR.95H=coxSummary$conf.int[,"upper .95"],

pvalue=coxSummary$coefficients[,"Pr(>|z|)"])

)

}

write.table(uniTab,file=uniOutFile,sep="\t",row.names=F,quote=F)

bioForest(coxFile=uniOutFile, forestFile=uniForest, forestCol="blue")

uniTab=uniTab[as.numeric(uniTab[,"pvalue"])<1,]

rt1=rt[,c("futime", "fustat", as.vector(uniTab[,"id"]))]

multiCox=coxph(Surv(futime, fustat) ~ ., data = rt1)

multiCoxSum=summary(multiCox)

multiTab=data.frame()

multiTab=cbind(

HR=multiCoxSum$conf.int[,"exp(coef)"],

HR.95L=multiCoxSum$conf.int[,"lower .95"],

HR.95H=multiCoxSum$conf.int[,"upper .95"],

pvalue=multiCoxSum$coefficients[,"Pr(>|z|)"])

multiTab=cbind(id=row.names(multiTab),multiTab)

write.table(multiTab,file=multiOutFile,sep="\t",row.names=F,quote=F)

bioForest(coxFile=multiOutFile, forestFile=multiForest, forestCol="red")

}

indep(riskFile="nomoRisk.txt",

cliFile="clinical.txt",

uniOutFile="uniCox.txt",

multiOutFile="multiCox.txt",

uniForest="uniForest.pdf",

multiForest="multiForest.pdf")

17

library("limma")

expFile="symbol.txt"

setwd("D:\\144fatty\\29.CIBERSORT")

rt=read.table(expFile, header=T, sep="\t", check.names=F)

rt=as.matrix(rt)

rownames(rt)=rt[,1]

exp=rt[,2:ncol(rt)]

dimnames=list(rownames(exp),colnames(exp))

data=matrix(as.numeric(as.matrix(exp)),nrow=nrow(exp),dimnames=dimnames)

data=avereps(data)

data=data[rowMeans(data)>0,]

v=voom(data, plot=F, save.plot=F)

out=v$E

out=rbind(ID=colnames(out), out)

write.table(out,file="uniq.symbol.txt",sep="\t",quote=F,col.names=F)

source("fatty29.CIBERSORT.R")

results=CIBERSORT("ref.txt", "uniq.symbol.txt", perm=1000, QN=TRUE)

18

library(limma)

library(reshape2)

library(ggpubr)

riskFile="risk.TCGA.txt"

immFile="CIBERSORT-Results.txt"

pFilter=0.05

setwd("D:\\144fatty\\30.immCor")

immune=read.table(immFile, header=T, sep="\t", check.names=F, row.names=1)

immune=immune[immune[,"P-value"]<pFilter,]

data=as.matrix(immune[,1:(ncol(immune)-3)])

group=sapply(strsplit(row.names(data),"\\-"), "[", 4)

group=sapply(strsplit(group,""), "[", 1)

group=gsub("2", "1", group)

data=data[group==0,]

row.names(data)=gsub("(.*?)\\-(.*?)\\-(.*?)\\-(.*?)\\-.*", "\\1\\-\\2\\-\\3", row.names(data))

data=avereps(data)

risk=read.table(riskFile, header=T, sep="\t", check.names=F, row.names=1)

sameSample=intersect(row.names(data), row.names(risk))

rt=cbind(data[sameSample,,drop=F], risk[sameSample,"Risk",drop=F])

data=rt[order(rt$Risk, decreasing=T),]

data=melt(data, id.vars=c("Risk"))

colnames(data)=c("Risk", "Immune", "Expression")

group=levels(factor(data$Risk))

data$Risk=factor(data$Risk, levels=c("low","high"))

bioCol=c("#0066FF","#FF0000","#6E568C","#7CC767","#223D6C","#D20A13","#FFD121","#088247","#11AA4D")

bioCol=bioCol[1:length(group)]

boxplot=ggboxplot(data, x="Immune", y="Expression", fill="Risk",

xlab="",

ylab="Fraction",

legend.title="Risk",

width=0.8,

palette=bioCol)+

rotate_x_text(50)+

stat_compare_means(aes(group=Risk),symnum.args=list(cutpoints=c(0, 0.001, 0.01, 0.05, 1), symbols=c("***", "**", "*", "ns")), label="p.signif")

pdf(file="immune.diff.pdf", width=8, height=6)

print(boxplot)

dev.off()

20

library(limma)

library(GSVA)

library(GSEABase)

library(ggpubr)

library(reshape2)

expFile="symbol.txt"

gmtFile="immune.gmt"

riskFile="risk.TCGA.txt"

setwd("D:\\144fatty\\31.immFunction")

rt=read.table(expFile, header=T, sep="\t", check.names=F)

rt=as.matrix(rt)

rownames(rt)=rt[,1]

exp=rt[,2:ncol(rt)]

dimnames=list(rownames(exp),colnames(exp))

mat=matrix(as.numeric(as.matrix(exp)),nrow=nrow(exp),dimnames=dimnames)

mat=avereps(mat)

mat=mat[rowMeans(mat)>0,]

geneSet=getGmt(gmtFile, geneIdType=SymbolIdentifier())

ssgseaScore=gsva(mat, geneSet, method='ssgsea', kcdf='Gaussian', abs.ranking=TRUE)

normalize=function(x){

return((x-min(x))/(max(x)-min(x)))}

data=normalize(ssgseaScore)

ssgseaOut=rbind(id=colnames(data), data)

write.table(ssgseaOut, file="immFunScore.txt", sep="\t", quote=F, col.names=F)

group=sapply(strsplit(colnames(data),"\\-"), "[", 4)

group=sapply(strsplit(group,""), "[", 1)

group=gsub("2", "1", group)

data=t(data[,group==0])

rownames(data)=gsub("(.*?)\\-(.*?)\\-(.*?)\\-(.*?)\\-.*", "\\1\\-\\2\\-\\3", rownames(data))

data=avereps(data)

risk=read.table(riskFile, header=T, sep="\t", check.names=F, row.names=1)

sameSample=intersect(row.names(data),row.names(risk))

data=data[sameSample,,drop=F]

risk=risk[sameSample,"Risk",drop=F]

rt1=cbind(data, risk)

data=melt(rt1, id.vars=c("Risk"))

colnames(data)=c("Risk","Type","Score")

data$Risk=factor(data$Risk, levels=c("low","high"))

p=ggboxplot(data, x="Type", y="Score", color = "Risk",

xlab="",ylab="Score",

palette = c("blue","red"), add = "none")

p=p+rotate_x_text(50)

p=p+stat_compare_means(aes(group=Risk),symnum.args=list(cutpoints = c(0, 0.001, 0.01, 0.05, 1), symbols = c("***", "**", "*", "ns")),label = "p.signif")

pdf(file="immFunction.pdf", width=8, height=6)

print(p)

dev.off()

21

library(limma)

library(GSEABase)

library(GSVA)

library(pheatmap)

expFile="symbol.txt"

riskFile="risk.TCGA.txt"

gmtFile="c2.cp.kegg.v7.4.symbols.gmt"

setwd("D:\\144fatty\\32.GSVA")

rt=read.table(expFile, header=T, sep="\t", check.names=F)

rt=as.matrix(rt)

rownames(rt)=rt[,1]

exp=rt[,2:ncol(rt)]

dimnames=list(rownames(exp), colnames(exp))

data=matrix(as.numeric(as.matrix(exp)), nrow=nrow(exp), dimnames=dimnames)

data=avereps(data)

geneSets=getGmt(gmtFile, geneIdType=SymbolIdentifier())

gsvaResult=gsva(data,

geneSets,

min.sz=10,

max.sz=500,

verbose=TRUE,

parallel.sz=1)

data=t(gsvaResult)

group=sapply(strsplit(row.names(data),"\\-"), "[", 4)

group=sapply(strsplit(group,""), "[", 1)

group=gsub("2", "1", group)

data=data[group==0,]

row.names(data)=gsub("(.*?)\\-(.*?)\\-(.*?)\\-(.*?)\\-.*", "\\1\\-\\2\\-\\3", row.names(data))

data=avereps(data)

risk=read.table(riskFile, header=T, sep="\t", check.names=F, row.names=1)

sameSample=intersect(row.names(data), row.names(risk))

data=data[sameSample,,drop=F]

risk=risk[sameSample,"Risk",drop=F]

gsvarisk=cbind(data, risk)

con=gsvarisk[gsvarisk$Risk=="low",]

treat=gsvarisk[gsvarisk$Risk=="high",]

data=rbind(con, treat)

Type=as.vector(data$Risk)

Type=factor(Type, levels=c("low", "high"))

ann=data[,ncol(data),drop=F]

data=t(data[,-ncol(data),drop=F])

design=model.matrix(~0+factor(Type))

colnames(design)=levels(factor(Type))

fit=lmFit(data, design)

cont.matrix=makeContrasts(high-low, levels=design)

fit2=contrasts.fit(fit, cont.matrix)

fit2=eBayes(fit2)

allDiff=topTable(fit2,adjust='fdr',number=200000)

allDiffOut=rbind(id=colnames(allDiff),allDiff)

write.table(allDiffOut, file="all.txt", sep="\t", quote=F, col.names=F)

diffSig=allDiff[with(allDiff, (abs(logFC)>0.1 & adj.P.Val < 0.05)), ]

diffSigOut=rbind(id=colnames(diffSig), diffSig)

write.table(diffSigOut, file="diff.txt", sep="\t", quote=F, col.names=F)

ann_colors=list()

bioCol=c("blue", "red")

names(bioCol)=c("low", "high")

ann_colors[["Risk"]]=bioCol

termNum=50

diffTermName=as.vector(rownames(diffSig))

diffLength=length(diffTermName)

if(diffLength<termNum){termNum=diffLength}

hmGene=diffTermName[1:termNum]

hmExp=data[hmGene,]

pdf(file="heatmap.pdf", width=10, height=6)

pheatmap(hmExp,

annotation=ann,

annotation_colors = ann_colors,

color = colorRampPalette(c(rep("blue",3), "white", rep("red",3)))(50),

cluster_cols=F,

show_colnames = F,

gaps_col=as.vector(cumsum(table(Type))),

scale="row",

fontsize = 7,

fontsize_row=6,

fontsize_col=7)

dev.off()

22

library(ggpubr)

riskFile="risk.TCGA.txt"

mutFile="mutMatrix.txt"

mutGene="TP53"

setwd("D:\\144fatty\\33.mutRisk")

risk=read.table(riskFile, header=T, sep="\t", check.names=F, row.names=1)

risk$riskScore[risk$riskScore>quantile(risk$riskScore,0.99)]=quantile(risk$riskScore,0.99)

mut=read.table(mutFile, header=T, sep="\t", check.names=F, row.names=1)

mut=t(mut[mutGene,,drop=F])

colnames(mut)=c("Type")

sameSample=intersect(row.names(mut), row.names(risk))

mut=mut[sameSample,,drop=F]

risk=risk[sameSample,,drop=F]

data=cbind(as.data.frame(risk), as.data.frame(mut))

data$Type=paste0(mutGene, " " , data$Type)

data$Type=factor(data$Type, levels=c(paste0(mutGene, " Wild"), paste0(mutGene, " Mutation")) )

group=levels(factor(data$Type))

comp=combn(group,2)

my_comparisons=list()

for(i in 1:ncol(comp)){my_comparisons[[i]]<-comp[,i]}

data1=data[,c("riskScore", "Type")]

boxplot=ggboxplot(data1, x="Type", y="riskScore", fill="Type",

xlab="",

ylab="Risk score",

legend.title="",

palette=c("blue2", "red2") )+

stat_compare_means(comparisons = my_comparisons)

pdf(file=paste0(mutGene, ".pdf"), width=5, height=4.5)

print(boxplot)

dev.off()

23

library(limma)

library(ggpubr)

library(pRRophetic)

library(ggplot2)

set.seed(12345)

pFilter=0.001

expFile="symbol.txt"

riskFile="risk.TCGA.txt"

setwd("D:\\144fatty\\34.pRRophetic")

data(cgp2016ExprRma)

data(PANCANCER_IC_Tue_Aug_9_15_28_57_2016)

allDrugs=unique(drugData2016$Drug.name)

rt = read.table(expFile, header=T, sep="\t", check.names=F)

rt=as.matrix(rt)

rownames(rt)=rt[,1]

exp=rt[,2:ncol(rt)]

dimnames=list(rownames(exp),colnames(exp))

data=matrix(as.numeric(as.matrix(exp)),nrow=nrow(exp),dimnames=dimnames)

data=avereps(data)

data=data[rowMeans(data)>0.5,]

group=sapply(strsplit(colnames(data),"\\-"), "[", 4)

group=sapply(strsplit(group,""), "[", 1)

group=gsub("2","1",group)

data=data[,group==0]

data=t(data)

rownames(data)=gsub("(.*?)\\-(.*?)\\-(.*?)\\-(.*)", "\\1\\-\\2\\-\\3", rownames(data))

data=avereps(data)

data=t(data)

riskRT=read.table(riskFile, header=T, sep="\t", check.names=F, row.names=1)

riskRT$riskScore[riskRT$riskScore>quantile(riskRT$riskScore,0.99)]=quantile(riskRT$riskScore,0.99)

for(drug in allDrugs){

possibleError=tryCatch(

{senstivity=pRRopheticPredict(data, drug, selection=1, dataset = "cgp2016")},

error=function(e) e)

if(inherits(possibleError, "error")){next}

senstivity=senstivity[senstivity!="NaN"]

senstivity[senstivity>quantile(senstivity,0.99)]=quantile(senstivity,0.99)

sameSample=intersect(row.names(riskRT), names(senstivity))

risk=riskRT[sameSample, c("riskScore","Risk"),drop=F]

senstivity=senstivity[sameSample]

rt=cbind(risk, senstivity)

rt$Risk=factor(rt$Risk, levels=c("low", "high"))

type=levels(factor(rt[,"Risk"]))

comp=combn(type, 2)

my_comparisons=list()

for(i in 1:ncol(comp)){my_comparisons[[i]]<-comp[,i]}

test=wilcox.test(senstivity~Risk, data=rt)

diffPvalue=test$p.value

x=as.numeric(rt[,"riskScore"])

y=as.numeric(rt[,"senstivity"])

corT=cor.test(x, y, method="spearman")

corPvalue=corT$p.value

if((diffPvalue<pFilter) & (corPvalue<pFilter)){

boxplot=ggboxplot(rt, x="Risk", y="senstivity", fill="Risk",

xlab="Risk",

ylab=paste0(drug, " senstivity (IC50)"),

legend.title="Risk",

palette=c("#0066FF","#FF0000")

)+

stat_compare_means(comparisons=my_comparisons)

pdf(file=paste0("durgSenstivity.", drug, ".pdf"), width=5, height=4.5)

print(boxplot)

dev.off()

df1=as.data.frame(cbind(x,y))

p1=ggplot(df1, aes(x, y)) +

xlab("Risk score") + ylab(paste0(drug, " senstivity (IC50)"))+

geom_point() + geom_smooth(method="lm",formula = y ~ x) + theme_bw()+

stat_cor(method = 'spearman', aes(x =x, y =y))

pdf(file=paste0("Cor.", drug, ".pdf"), width=5, height=4.6)

print(p1)

dev.off()

}

}

24

library(limma)

library(ggpubr)

tideFile="TIDE.txt"

riskFile="risk.TCGA.txt"

setwd("D:\\144fatty\\35.TIDE")

tide=read.table(tideFile, header=T, sep="\t", check.names=F, row.names=1)

group=sapply(strsplit(row.names(tide),"\\-"), "[", 4)

group=sapply(strsplit(group,""), "[", 1)

group=gsub("2", "1", group)

tide=tide[group==0,,drop=F]

row.names(tide)=gsub("(.*?)\\-(.*?)\\-(.*?)\\-(.*?)\\-.*", "\\1\\-\\2\\-\\3", row.names(tide))

tide=avereps(tide)

risk=read.table(riskFile, header=T, sep="\t", check.names=F, row.names=1)

sameSample=intersect(row.names(tide), row.names(risk))

tide=tide[sameSample, , drop=F]

risk=risk[sameSample, "Risk", drop=F]

data=cbind(tide, risk)

data$Risk=ifelse(data$Risk=="high", "High-risk", "Low-risk")

group=levels(factor(data$Risk))

data$Risk=factor(data$Risk, levels=c("Low-risk", "High-risk"))

group=levels(factor(data$Risk))

comp=combn(group,2)

my_comparisons=list()

for(i in 1:ncol(comp)){my_comparisons[[i]]<-comp[,i]}

gg1=ggviolin(data, x="Risk", y="TIDE", fill = "Risk",

xlab="", ylab="TIDE",

palette=c("#0066FF","#FF0000"),

legend.title="Risk",

add = "boxplot", add.params = list(fill="white"))+

stat_compare_means(comparisons = my_comparisons,symnum.args=list(cutpoints = c(0, 0.001, 0.01, 0.05, 1), symbols = c("***", "**", "*", "ns")),label = "p.signif")

pdf(file="TIDE.pdf", width=6, height=5)

print(gg1)

dev.off()

25

library(limma)

expFile="symbol.txt"

riskFile="risk.TCGA.txt"

logFCfilter=1

fdrFilter=0.05

setwd("D:\\144fatty\\36.riskDiff")

rt=read.table(expFile, header=T, sep="\t", check.names=F)

rt=as.matrix(rt)

rownames(rt)=rt[,1]

exp=rt[,2:ncol(rt)]

dimnames=list(rownames(exp), colnames(exp))

data=matrix(as.numeric(as.matrix(exp)), nrow=nrow(exp), dimnames=dimnames)

data=avereps(data)

group=sapply(strsplit(colnames(data),"\\-"), "[", 4)

group=sapply(strsplit(group,""), "[", 1)

group=gsub("2", "1", group)

data=data[,group==0]

data=t(data)

rownames(data)=gsub("(.*?)\\-(.*?)\\-(.*?)\\-(.*?)\\-.*", "\\1\\-\\2\\-\\3", rownames(data))

data=avereps(data)

data=t(data)

risk=read.table(riskFile, header=T, sep="\t", check.names=F, row.names=1)

sameSample=intersect(colnames(data), row.names(risk))

data=data[,sameSample]

risk=risk[sameSample,]

riskLow=risk[risk$Risk=="low",]

riskHigh=risk[risk$Risk=="high",]

dataLow=data[,row.names(riskLow)]

dataHigh=data[,row.names(riskHigh)]

data=cbind(dataLow,dataHigh)

data=data[rowMeans(data)>1,]

conNum=ncol(dataLow)

treatNum=ncol(dataHigh)

Type=c(rep(1,conNum), rep(2,treatNum))

outTab=data.frame()

for(i in row.names(data)){

rt=data.frame(expression=data[i,], Type=Type)

wilcoxTest=wilcox.test(expression ~ Type, data=rt)

conGeneMeans=mean(data[i,1:conNum])

treatGeneMeans=mean(data[i,(conNum+1):ncol(data)])

logFC=log2(treatGeneMeans)-log2(conGeneMeans)

pvalue=wilcoxTest$p.value

conMed=median(data[i,1:conNum])

treatMed=median(data[i,(conNum+1):ncol(data)])

diffMed=treatMed-conMed

if( ((logFC>0) & (diffMed>0)) | ((logFC<0) & (diffMed<0)) ){

outTab=rbind(outTab,cbind(gene=i,lowMean=conGeneMeans,highMean=treatGeneMeans,logFC=logFC,pValue=pvalue))

}

}

pValue=outTab[,"pValue"]

fdr=p.adjust(as.numeric(as.vector(pValue)), method="fdr")

outTab=cbind(outTab, fdr=fdr)

outDiff=outTab[( abs(as.numeric(as.vector(outTab$logFC)))>logFCfilter & as.numeric(as.vector(outTab$fdr))<fdrFilter),]

write.table(outDiff, file="riskDiff.txt", sep="\t", row.names=F, quote=F)

write.table(outDiff, file="riskDiff.xls", sep="\t", row.names=F, quote=F)

26

library("clusterProfiler")

library("org.Hs.eg.db")

library("enrichplot")

library("ggplot2")

library(GOplot)

pvalueFilter=0.05

qvalueFilter=0.05

colorSel="qvalue"

if(qvalueFilter>0.05){

colorSel="pvalue"

}

setwd("D:\\144fatty\\37.GO")

rt=read.table("diff.txt", header=T, sep="\t", check.names=F)

genes=as.vector(rt[,1])

entrezIDs=mget(genes, org.Hs.egSYMBOL2EG, ifnotfound=NA)

entrezIDs=as.character(entrezIDs)

gene=entrezIDs[entrezIDs!="NA"]

#gene=gsub("c\\(\"(\\d+)\".*", "\\1", gene)

kk=enrichGO(gene=gene,OrgDb=org.Hs.eg.db, pvalueCutoff=1, qvalueCutoff=1, ont="all", readable =T)

GO=as.data.frame(kk)

GO=GO[(GO$pvalue<pvalueFilter & GO$qvalue<qvalueFilter),]

write.table(GO,file="GO.txt",sep="\t",quote=F,row.names = F)

showNum=10

if(nrow(GO)<30){

showNum=nrow(GO)

}

pdf(file="barplot.pdf", width=8, height=7)

bar=barplot(kk, drop = TRUE, showCategory =showNum,split="ONTOLOGY",label_format=50, color = colorSel) + facet_grid(ONTOLOGY~., scale='free')

print(bar)

dev.off()

27

library("clusterProfiler")

library("org.Hs.eg.db")

library("enrichplot")

library("ggplot2")

library(GOplot)

pvalueFilter=0.05

qvalueFilter=0.05

colorSel="qvalue"

if(qvalueFilter>0.05){

colorSel="pvalue"

}

setwd("D:\\144fatty\\38.KEGG")

rt=read.table("riskDiff.txt", header=T, sep="\t", check.names=F)

genes=as.vector(rt[,1])

entrezIDs=mget(genes, org.Hs.egSYMBOL2EG, ifnotfound=NA)

entrezIDs=as.character(entrezIDs)

rt=cbind(rt,entrezID=entrezIDs)

gene=entrezIDs[entrezIDs!="NA"]

#gene=gsub("c\\(\"(\\d+)\".*", "\\1", gene)

kk <- enrichKEGG(gene=gene, organism="hsa", pvalueCutoff=1, qvalueCutoff=1)

KEGG=as.data.frame(kk)

KEGG$geneID=as.character(sapply(KEGG$geneID,function(x)paste(rt$gene[match(strsplit(x,"/")[[1]],as.character(rt$entrezID))],collapse="/")))

KEGG=KEGG[(KEGG$pvalue<pvalueFilter & KEGG$qvalue<qvalueFilter),]

write.table(KEGG, file="KEGG.txt", sep="\t", quote=F, row.names = F)

showNum=30

if(nrow(KEGG)<showNum){

showNum=nrow(KEGG)

}

pdf(file="barplot.pdf", width=8, height=7)

barplot(kk, drop = TRUE, showCategory = showNum, label_format=50, color = colorSel)

dev.off()

pdf(file="bubble.pdf", width=8, height=7)

dotplot(kk, showCategory = showNum, orderBy = "GeneRatio", label_format=50, color = colorSel)

dev.off()

kegg=data.frame(Category="ALL", ID = KEGG$ID, Term=KEGG$Description, Genes = gsub("/", ", ", KEGG$geneID), adj_pval = KEGG$p.adjust)

genelist <- data.frame(ID = rt$gene, logFC = rt$logFC)

row.names(genelist)=genelist[,1]

circ <- circle_dat(kegg, genelist)

termNum =8

termNum=ifelse(nrow(kegg)<termNum,nrow(kegg),termNum)

geneNum=300

geneNum=ifelse(nrow(genelist)<geneNum, nrow(genelist), geneNum)

chord <- chord_dat(circ, genelist[1:geneNum,], kegg$Term[1:termNum])

pdf(file="KEGGcircos.pdf", width=11, height=11)

GOChord(chord,

space = 0.001,

gene.order = 'logFC',

gene.space = 0.25,

gene.size = 5,

border.size = 0.1,

process.label = 6)

dev.off()
